# Diet and Physical Activity Interventions to Prevent or Treat Obesity in South Asian Children and Adults: A Systematic Review and Meta-Analysis 

**DOI:** 10.3390/ijerph120100566

**Published:** 2015-01-09

**Authors:** Tamara Brown, Sarah Smith, Raj Bhopal, Adetayo Kasim, Carolyn Summerbell

**Affiliations:** 1 Obesity Related Behaviours (ORB) Research Group, School of Medicine, Pharmacy and Health, Wolfson Research Institute for Health and Wellbeing, Queen’s Campus, Durham University, Stockton-on-Tees, TS17 6BH, UK; E-Mails: sarah.smith@durham.ac.uk (S.S.); carolyn.summerbell@durham.ac.uk (C.S.); 2 Edinburgh Ethnicity and Health Research Group, Centre for Population Health Sciences, University of Edinburgh, Teviot Place, Edinburgh, EH89AG, UK; E-Mail: raj.bhopal@ed.ac.uk; 3 Wolfson Research Institute for Health and Wellbeing, Queen’s Campus, Durham University, Stockton-on-Tees TS17 6BH, UK; E-Mail: a.s.kasim@durham.ac.uk

**Keywords:** obesity, South Asian, systematic review, children, adults, diet, physical activity

## Abstract

*Background and Aims*: The metabolic risks associated with obesity are greater for South Asian populations compared with White or other ethnic groups, and levels of obesity in childhood are known to track into adulthood. Tackling obesity in South Asians is therefore a high priority. The rationale for this systematic review is the suggestion that there may be differential effectiveness in diet and physical activity interventions in South Asian populations compared with other ethnicities. The research territory of the present review is an emergent, rather than mature, field of enquiry, but is urgently needed. Thus the aim of this systematic review and meta-analysis was to assess the effectiveness of diet and physical activity interventions to prevent or treat obesity in South Asians living in or outside of South Asia and to describe the characteristics of effective interventions. *Methods*: Systematic review of any type of lifestyle intervention, of any length of follow-up that reported any anthropometric measure for children or adults of South Asian ethnicity. There was no restriction on the type of comparator; randomised controlled trials, controlled clinical trials, and before-after studies were included. A comprehensive search strategy was implemented in five electronic databases: ASSIA, Cochrane Controlled Trials Register, Embase, Medline and Social Sciences Citation Index. The search was limited to English language abstracts published between January 2006 and January 2014. References were screened; data extraction and quality assessment were carried out by two reviewers. Results are presented in narrative synthesis and meta-analysis. *Results*: Twenty-nine studies were included, seven children, 21 adult and one mixed age. No studies in children under six were identified. Sixteen studies were conducted in South Asia, ten in Europe and three in USA. Effective or promising trials include physical activity interventions in South Asian men in Norway and South Asian school-children in the UK. A home-based, family-orientated diet and physical activity intervention improved obesity outcomes in South Asian adults in the UK, when adjusted for baseline differences. Meta-analyses of interventions in children showed no significant difference between intervention and control for body mass index or waist circumference. Meta-analyses of adult interventions showed significant improvement in weight in data from two trials adjusted for baseline differences (mean difference −1.82 kgs, 95% confidence interval −2.48 to −1.16) and in unadjusted data from three trials following sensitivity analysis (mean difference −1.20 kgs, 95% confidence interval −2.23 to −0.17). Meta-analyses showed no significant differences in body mass index and waist circumference for adults. Twenty of 24 intervention groups showed improvements in adult body mass index from baseline to follow-up; average change in high quality studies (*n* = 7) ranged from 0.31 to −0.8 kg/m^2^. There was no evidence that interventions were more or less effective according to whether the intervention was set in South Asia or not, or by socio-economic status. *Conclusions*: Meta-analysis of a limited number of controlled trials found an unclear picture of the effects of interventions on body mass index for South Asian children. Meta-analyses of a limited number of controlled trials showed significant improvement in weight for adults but no significant differences in body mass index and waist circumference. One high quality study in South Asian children found that a school-based physical activity intervention that was delivered within the normal school day which was culturally sensitive, was effective. There is also evidence of culturally appropriate approaches to, and characteristics of, effective interventions in adults which we believe could be transferred and used to develop effective interventions in children.

## 1. Introduction

Persistently high levels of childhood and adulthood overweight and obesity globally, and the associated health complications, have been well documented [1,2,3]. In high income countries, evidence from epidemiological studies have shown inconsistent relationships in the prevalence of obesity in immigrant South Asian children and adults compared with native White populations [4]. However, the metabolic risks associated with obesity are greater for South Asian populations compared with White or other ethnic groups [5,6]. Given that levels of obesity in childhood are known to track into adulthood [7] tackling obesity in South Asians is therefore a high priority for public health in terms of reducing ethnic inequalities. 

There is also evidence that, outside of South Asia, universal health promoting interventions are taken up more effectively by White compared with South Asian populations and therefore widen ethnic inequalities in obesity even further [8]. Although there is reasonable evidence that access and recruitment to universal interventions is a significant issue for South Asians populations, and can contribute to health inequalities [9], Bhopal [10] also argues that approaches to, and characteristics of, the intervention itself can contribute to differential effectiveness. Differential effectiveness may arise at a number of points in the implementation of an intervention, including intervention efficacy, service provision or access, uptake, and compliance [11]. A common assumption is that health promotion interventions found to be effective in the general population are also, if appropriately adapted, likely to prove effective when targeted at ethnic minority populations, but there is limited evidence available on which to judge this to be true or not [8]. 

There is a substantial body of theoretical work and guidance on the kinds of interventions which are likely to reduce or increase health inequalities [12,13,14] and Lorenc [15] has conducted a rapid overview of systematic reviews to identify the types of interventions that are more likely to produce differential effects, and which have the potential to reduce inequalities. Lorenc [15] concluded that their findings are consistent with the idea that “downstream” preventative interventions are more likely to increase health inequalities than “upstream” interventions. Public health interventions can usefully be understood across an upstream-downstream continuum within a framework proposed by Keleher [16]:

- Downstream interventions are those focused on change or support for individuals and include primary prevention. Examples include communication strategies, health information and behaviour change campaigns.
- Midstream interventions are those that focus on psychosocial levels and behaviours. Midstream interventions include social marketing and the provision of health education to individuals, communities and populations more broadly. Community action and community development interventions, which often encompass awareness raising, are also directed at social change, so they are sometimes considered to be more upstream than midstream.
- Upstream interventions take a population focus, and are also intended as change mechanisms, to support efforts to promote social change. Upstream interventions encompass institutional practices and systems change, organisational change and development, workforce development, policy, and legislation, to influence social (including ethnic) norms that create and reinforce social and health inequalities.

It is also possible that the way in which a complex intervention is organised and implemented (*i.e.*, context) can impact on its ability to reduce ethnic inequalities [17]. For example, a recent systematic review by Durand [18] suggests that interventions which involve shared decision making (and increase participant engagement) may be more beneficial to disadvantaged groups. Nearly all systematic reviews only examine the effects of interventions that reduce overall levels of obesity, although we and others have recently published systematic reviews on the effects on socioeconomic inequalities in obesity [19,20]. To the best of our knowledge, there is a lack of accessible policy ready evidence on what works in terms of interventions to reduce ethnic inequalities in childhood and adulthood obesity. 

There is evidence from trials that some diet and physical activity interventions can effectively prevent or treat obesity in children [21] and adults [22] but most of these trials have been conducted in predominately White populations or targeted at African Americans. The research territory of the present review is an emergent, rather than mature, field of enquiry, but is urgently needed. Given the dearth of relevant trial evidence in South Asian populations [23], particularly South Asian children [24], the aim of this systematic review was to assess the effectiveness of diet and physical activity interventions to prevent or treat obesity in South Asian children and adults and to describe the characteristics of effective interventions. Initially this research was focused entirely on South Asian children [24] but given the paucity of the data the review was redesigned to be more inclusive and include all South Asian populations.

## 2. Methods

The methods of the review are registered with the International Prospective Register of Systematic Reviews (PROSPERO registration no. CRD42014008800) [25]. The review was written following the Preferred Reporting Items for Systematic Reviews and Meta-Analyses (PRISMA) reporting guidelines [26]. 

### 2.1. Search Strategy and Study Selection 

A comprehensive search strategy was implemented in five electronic databases: ASSIA, Cochrane Controlled Trials Register, Embase, Medline and Social Sciences Citation Index. The search was limited to English language abstracts; published between January 2006 and January 2014. The search was started in January 2006 because two of the authors (Carolyn Summerbell and Tamara Brown) searched for the same evidence from 1990 to December 2005 as part of the supporting work for the NICE obesity guidance [27]. This previous search found only one underpowered uncontrolled 6-week study [28] which was published in 1999. Terms for South Asian ethnicity, diet and physical activity, and obesity, were combined using database specific terms and keywords (Figure S1). In addition to the database searches, three key reviews [23,24,29] and reference lists of screened full-text articles were also searched for relevant primary studies. 

A database of references generated from the search was produced using Reference Manager 12. The titles and abstracts plus full-text articles were screened by one reviewer (Tamara Brown). In cases where study inclusion was unclear, another reviewer was consulted (Carolyn Summerbell). Authors were contacted for clarification of study eligibility for inclusion, where necessary.

### 2.2. Eligibility Criteria

Based on previous reviews [23,24,29] it was anticipated that few randomised controlled trials (RCTs) would be identified. Therefore, to provide information on the characteristics of, and approaches to, potentially effective diet (D) and physical activity (PA) interventions to prevent or treat obesity in South Asians, the inclusion criteria were designed to be relatively broad. There was no restriction on the type of comparator used in the study; RCTs, controlled clinical trials (CCTs) and before-after studies (BAs) were included. Participants of any age were included, on the basis that cultural elements of an effective intervention in South Asian adults may be relevant to interventions in South Asian children, particularly young children where the parent is the main point of contact for recruitment and retention. Any type of lifestyle intervention (including D and/or PA), of any length of follow-up, that reported any anthropometric measure for children or adults of South Asian ethnicity, regardless of health status, was included. The intervention could be aimed at managing obesity or obesity-related diseases such as type 2 diabetes. Interventions were excluded if they focused on food supplementation, fortification, or complementary feeding; the prevention or treatment of undernutrition; eating disorders; surgery or drug treatment. South Asian ethnicity was defined as someone of South Asian origin with ancestry, parentage or birthplace in India, Pakistan, Bangladesh or Sri Lanka [30]. Studies that focussed exclusively on South Asians populations and studies that reported outcomes for a South Asian subgroup were included.

### 2.3. Data Extraction and Quality Assessment

Data were extracted by two reviewers (SS, TB) who checked each other’s extraction of outcome data. Data relating to study design, population characteristics, intervention details, and obesity outcomes were extracted. Details about how interventions were tailored and delivered (characteristics and approaches) for South Asian populations were also extracted. Primary outcomes extracted were: body mass index (BMI), body mass index z-score (zBMI), waist circumference (WC) and weight (WT); from our earlier review on the same topic [24] it was anticipated that these measures would be the most widely reported. Primary outcomes were extracted for South Asian groups and also by socio-economic status (SES) within South Asian groups. Key information from process evaluations were extracted, including development and implementation; compliance; fidelity; acceptability; and sustainability of interventions. Other information extracted included funding source and economic evaluations.

The methodological quality of the studies was assessed by one reviewer (TB) using the Six Item Checklist Of Quality Of Execution [31,32] adapted from the Effective Public Health Practice Project Quality Assessment Tool for Quantitative Studies [33]. This tool was chosen because it was developed to assess public health interventions; it enables reviewers to quality assess and synthesise evidence from a range of study designs and it is a widely used, valid and reliable tool. Quality assessment included representativeness of study samples; randomisation; baseline comparability of groups; credibility of data collection tools; attrition rate; and attributability. Studies were classified according to number of quality criteria achieved: “low” quality (0–2); “medium” quality (3–4) and “high” quality (5–6). Not all studies could achieve the maximum (6) because some criteria were not relevant to all study designs. Grouping of studies by quality does not imply that all quality criteria were equal; studies were grouped to provide a simple method of comparing quality between studies. Pertinent qualitative findings are discussed alongside study results.

### 2.4. Data Analysis

Results for all studies are presented as a narrative synthesis; eligible studies were also included in meta-analyses using Review Manager 5.2. Data were subgrouped by age and study design.

Effectiveness was assessed for each primary outcome (BMI/zBMI, WC, and WT) with a summary of overall effectiveness for each study. An intervention was classified as: effective (↑); equally effective/not effective (↔); effectiveness mixed by outcome or gender (**↕)**; not effective (↓) or effectiveness unclear (?). Where possible effectiveness was assessed using between group differences, otherwise effectiveness was assessed using within group differences from baseline to last follow-up. RCTs classified as “equally effective/not effective (↔)” were classified as such when there was no significant difference in effect between intervention and control groups; both groups could have demonstrated significant improvement or both groups could have shown no improvement from baseline to follow-up.

We conducted meta-analyses (random effect models) to determine change in outcomes from baseline to last follow-up, using mean differences (MDs) for adults and standardised mean differences (SMD) for children. Some studies adjusted outcome data for corresponding baseline differences between intervention and control groups; some of these studies also adjusted for other variables such as age, sex and ethnicity. In order to synthesise all available evidence we extracted both unadjusted and adjusted mean differences using the inverse variance data for adults, although we consider the baseline adjusted analysis as the definitive analysis for individual trial data. Data for children were standardised to incorporate all studies that reported a mix of BMI and zBMI; this meant that we were unable to synthesise adjusted and unadjusted data for children within the meta-analysis (cannot standardise adjusted mean difference data); the adjusted data are reported separately within the text. We planned to explore any heterogeneity through sensitivity analyses of study characteristics. 

In cases where both BMI and zBMI are reported within a study, zBMI was entered into the meta-analysis as this measure is standardised for age and gender using a reference population. Where reported, adjusted and unadjusted mean differences (for adults) were extracted directly into Review Manager. Otherwise, unadjusted mean differences were obtained using continuous data derived by extracting mean change and standard deviation (SD) from baseline to last follow-up for intervention and control groups. When necessary, change was calculated by subtracting outcome values at the last follow-up from the corresponding baseline value and the SD was imputed from baseline. The I2 statistic was used to measure heterogeneity. Results with P > 0.05 were reported as not significant.

## 3. Results

**Figure 1:**
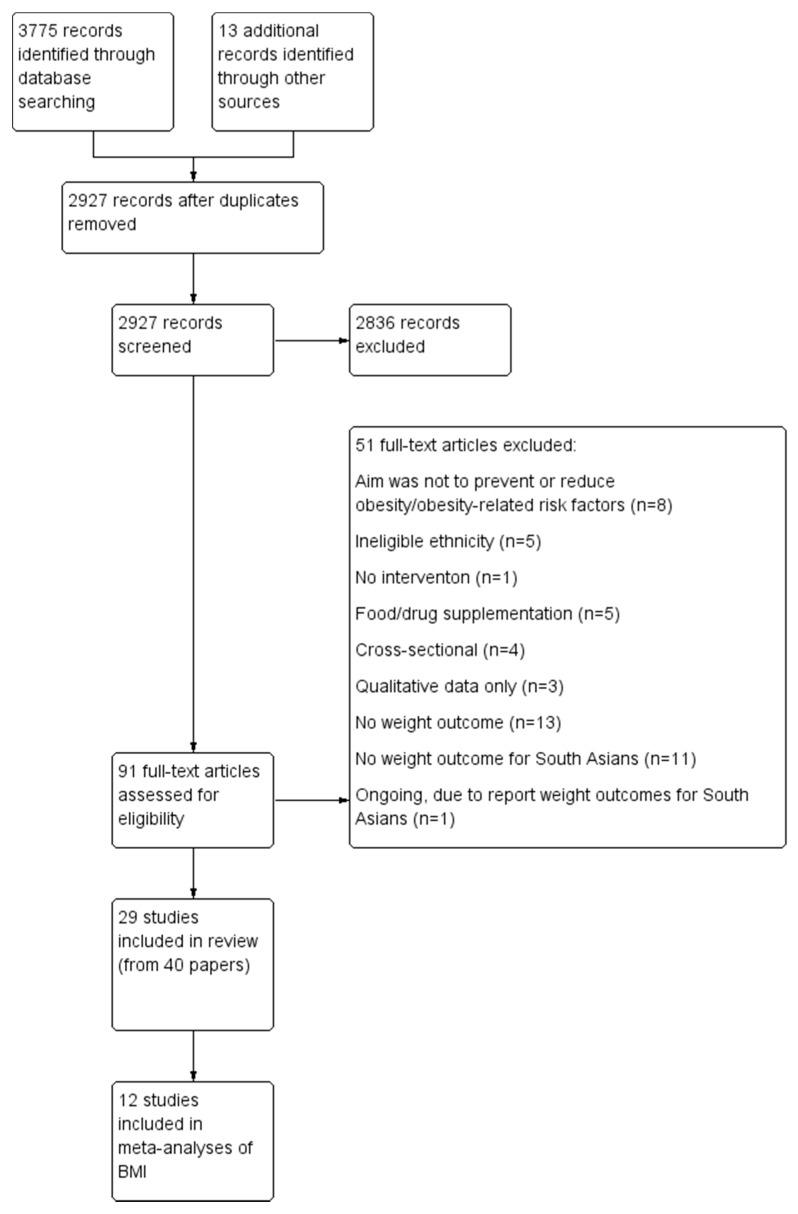
Study flow.

The electronic search produced 3775 records and an additional 13 records were identified through other sources (Figure 1). Other sources included key reviews, Google, reference lists of included papers, and contacting experts. After removing duplicate records, 2927 titles and abstracts were screened. Ninety-one full-text articles were assessed; 29 studies (from 40 papers) were included and 51 articles were excluded.

### 3.1. Methodological Characteristics and Quality of Included Studies

Twenty-nine studies [34,35,36,37,38,39,40,41,42,43,44,45,46,47,48,49,50,51,52,53,54,55,56,57,58,59,60,61,62] were included, with 11 additional linked papers [63,64,65,66,67,68,69,70,71,72,73]. 10 studies were RCTs, two CCTs, and 17 studies were classified as BAs. Study characteristics are summarised in Table 1. In some cases not all groups within a study met the review inclusion criteria; only data relating to the included group(s) were extracted and quality assessed. For example, two studies [43,45] were classified as BAs but were, in fact, dietary control groups from RCTs (where the intervention group was not eligible for inclusion). Another study [61] was classified as a BA because we derived data from a subset cohort of participants that were assessed at both baseline and follow-up; the cohort was nested within a larger cross-sectional study design. Only three of the BAs had more than one study group that were eligible for inclusion [57,58,61].

All 29 studies were assessed for quality using six factors (representativeness, randomisation, comparability, credibility, attrition, and attributability; Table S1 contains full details by study). Only nine studies had study samples that were deemed to be a relatively unbiased group of the specific population from which they represented [36,38,39,51,54,56,59,60,61]. Of the 15 studies that could be assessed for comparability between study groups on various socioeconomic and outcome variables at baseline, 10 were deemed to have comparable study groups. 25 studies used valid and reliable data collection tools. Six studies had unacceptably high attrition rates (30% and above). In seven studies it was not possible to be confident that the observed effects were attributable to the intervention. 14 studies were based in India, five in UK, three in USA, two in Pakistan, two in Norway, and one each in Australia, Netherlands and New Zealand (see Table S2 for detailed study characteristics). There were 11 controlled trials (CCTs/RCTs) of which five were based in South Asian countries and six in non-South Asian countries. There were seven studies exclusively of children (mean age ranging from 6 to 16 years) and 21 studies exclusively of adults (mean age ranging from 31 to 62 years). One study included both children and adults [38]. Two studies [46,50], both of children, included other ethnicities and reported outcomes separately for subgroups of South Asian children comprising approximately 25% of the total samples. BEACHeS included 86% South Asian children and reported on the total sample [62].

Eleven adult studies set a minimum BMI as part of study inclusion criteria. Across all adult studies BMI ranged from 21 to 36 kg/m^2^. The majority of studies had a baseline BMI which ranged from 26 to 31 kg/m^2^. In some studies of the general population, the participants were overweight but otherwise generally healthy, and in other studies, as would be expected, there were a proportion of participants that had obesity-related co-morbidities including type 2 diabetes, metabolic syndrome and risk factors for cardiovascular disease. Some studies targeted adults with co-morbidities, including adults with or at risk of diabetes; with hypertension; with or at risk of metabolic syndrome, infertile women; and adolescent girls with polycystic ovary syndrome. 

Participant numbers ranged from 23 to 2331. Study duration ranged from three to 48 months for adult interventions. With the exception of one study which had a 24-month follow-up [46] children’s interventions ranged from three to eight months. Settings included general practice, community, home, hospital, school, university and workplace. Types of interventions included were: D (5), PA (6) and combined D and PA (18). Unique interventions included a dietary intervention based on the Ayurvedic constitution [58] and a yoga-based intervention [53]. 

**Table 1:** Study characteristics.

Study ID	No.	I % Male	C % Male	I Age (Years)	C Age (Years)	Ethnicity	Min BMI *	I Mean Baseline BMI	C Mean Baseline BMI	Target Behaviour Change	Country	Setting	Duration Months
**RCTs (children)**																									
Singhal 2010 [59]	201	61	60	16	16	Asian Indian	N	NR	NR	D&PA	India	School	6
Nidhi 2012 [53]	90	0	0	16	16	Asian Indian	N	20.3	21.2	PA	India	School	3
Johnston 2013 [46]	835	62	54	8	8	25% Asian	N	21.6	21.0	D&PA	USA	School	24
**CCTs**																									
Almas 2013 [35]	280	0	0	10	10	Pakistani	N	1.35 (z-score)	1.92 (z-score)	PA	Pakistan	School	5
Adab 2014 [62]	574	54	50	7	6	86% South Asian	N	−0.03 (z-score)	0.08 (z-score)	D&PA	UK	School	4
**BA (children)**																									
Balagopal 2008 [38] **	118	48	NA	14	NA	Asian Indian	N	16.0	NA	D&PA	India	Community	7
Kameswararao 2009 [47] **^a^**	59	61	NA	School age	School age	Asian Indian	Y	59/610 BMI ≥ 95th percentile	NA	D&PA	India	School	6
Madsen 2009 [50]	233	52	NA	10	NA	26% South Asian	N	20.9	NA	PA	USA	Community	8
**RCTs (adults)**																									
Ramachandran 2006 [54] **^b^**	269	78	76	46	45	Asian Indian	N	25.7	26.3	D&PA	India	Community	30
Bellary 2008 [40]	1486	54	49	57	57	South Asian	N	28.5	28.6	D	UK	General Practice	24
Admiraal 2013 [34]	536	50	51	45	45	Hindustani Surinamese	N	28.1	27.2	D&PA	Netherlands	General Practice	12
Andersen 2013 [36]	150	100	100	36	40	Born/parents born in Pakistan	N	27.1	27.4	PA	Norway	Community University	5
Ramachandran 2013 [55]	537	100	100	46	46	Asian Indian	Y	25.8	25.8	D&PA	India	Workplace	20
Telle-Hjellset 2013 [60]	198	0	0	41	42	Born/both parents born in Pakistan	N	29.4	29.8	D&PA	Norway	Mother & child health clinic	7
Bhopal 2014 [41]	156	46	45	53	52	Indian, Pakistani origin	Y	30.6	30.5	D&PA	UK	Home	36
**BA (adults)**																									
Ghosh 2006 [44]	45	100	NA	60	NA	Asian Indian	Y	26.3	NA	PA	India	University	5
Mathews 2007 [51]	304	34	NA	44	NA	Bangladeshi Indian Pakistani	N	28.6	NA	D&PA	UK	Clinic, community	6–12
Rush 2007 [56]	41	53	NA	M: 62; F: 59	NA	Asian Indian	N	NR	NA	D&PA	New Zealand	Community, laboratory	5
Backes 2008 [37]	23	0	NA	44	NA	South Asian	Y	30.2	NA	D	USA	University	3
Balagopal 2008 [38] **	585	41	NA	40	NA	Asian Indian	N	20.6	NA	D&PA	India	Community	7
Dixon 2008 [43] **^c^**	22	53	NA	>25	NA	South Asian	Y	27.3	NA	D	UK	Hospital	12
Kousar 2008 [49]	53	0	NA	38	NA	Born in Pakistan	Y	29.2	NA	D&PA	Australia	University	6
Misra 2008 [52]	30	73	NA	41	NA	Asian Indian	N	24.1	NA	PA	India	Physiotherapy clinic	3
Prabhakaran 2009 [61] **^d^**	2331	60	57	41	39	Asian Indian	N	NR	NR	D&PA	India	Worksite	44–48
Sharma 2009 [58] **^e^**	200	12	12	20–60	20–60	Asian Indian	Y	Vatta 31.0 Pitta 32.0 Kapha 31.6	NA	D	India	Clinic	3
Shailaja 2011 [57] **^d^**	200	UM:81 UF:19 RM:46 RF:17	NA	18–60	18–60	Asian South Indian	Y	UM:28.1 UF:29.3 RM:27.1 RF:28.2	NA	D&PA	India	Community	3
Balagopal 2012 [39]	1681	46	NA	42	NA	Asian Indian	N	20.8	NA	D&PA	India	Community	6
Chander 2013 [42]	157	71	NA	60	NA	Asian Indian	N	30.6	NA	D&PA	India	Hospital	10
Khaskheli 2013 [48]	98	0	NA	31	NA	Pakistani	Y	36.2	NA	D&PA	Pakistan	Private clinic	12
Gulati 2014 [45] **^c^**	35	40	NA	43	NA	Asian Indian	Y	30.9	NA	D	India	Hospital	6

**^a^** obesity arm only; **^b^** lifestyle *vs.* control groups only; **^c^** diet group only; **^d^** 2 groups; **^e^** 3 groups; * minimum BMI stated as part of study inclusion criteria; ** reports outcomes for children and adults; BA: before-after studies; C: control group; CCT: controlled clinical trial; D: diet; F: female; I: intervention group; M: male; PA: physical activity; R: rural; RCT: randomised controlled trial; RF: rural female; RM: rural male; U: urban; UF: urban female; UM: urban male.

Examples of how dietary interventions were tailored included culturally adapted and translated resources such as booklets, a focus on locally available and low-cost fruit and vegetables; and cooking demonstrations adapting traditional ingredients and meals. Examples of how PA interventions were tailored included female-only groups with PA which did not include dance or music [35]. One PA intervention provided childcare, baby-friendly walking paths and walking shoes [60]. Some studies involved community participants in all aspects of the intervention including selection of community health workers. Those who delivered the interventions included linkworkers, people who spoke the same language, were the same sex, or from the same community as participants. The importance of involving other family members was recognised in many studies, particularly when making dietary changes.

Eleven studies reported using a theoretical framework; three studies were based on the stages of change construct from the transtheoretical model [51,55,60] and two studies applied the social cognitive theory of behaviour change [36,61]. One study used a community-based participatory research approach [39] and two studies used motivational interviewing techniques [34,46]. Other theories included cultural competence and peer education models, [49] psychosocial model [35] and the theory of Ayurvedic constitution [58]. BEACHeS [62,73] used the theoretical phase of the Medical Research Council (MRC) framework to develop the intervention and the Analysis Grid for Environments Linked to Obesity (ANGELO) framework which takes into account sociocultural influences on obesity. BEACHeS reported using an approach similar to a Theories of Change approach.

Twenty-one studies reported details of funding, of which 15 were non-industry funded and six were industry funded [40,43,45,52,54] including one study that was funded by a mix of public, corporate and individual donors [50]. Only three studies reported on the role of funders; reporting that funders had no role in study evaluation [40,41,55]. Of the six industry funded studies, three reported no conflict of interest [40,43,45] and three studies failed to report on conflict of interest [50,52,54]. Another study [58] did not report details of funding however the author is managing director of a private weight management company.

### 3.2. Effects of Interventions

Study outcomes are summarised in Table 2. See Table S3 for individual results for all studies including details of adjustments. Given the limited number of studies, the fact that all five controlled studies of children used completer analyses, and the presence of heterogeneity in outcome results, it was not possible to assess the effect of the type of outcome analysis on the results for children. Five of the adult RCTs performed completer analyses [34,36,41,54,60] and two performed intention-to-treat (ITT) analyses [40,55]. In one study [41] the dropout was minimal (2%) reducing the potential for attrition bias; in another study [34] the overall dropout was 38%, increasing the potential for attrition bias. Bellary [40] and Ramachandran [55] imputed outcome data using the Last Observation Carried Forward (LOCF) method; there was 14% dropout in the study by Bellary [40] and 4% dropout in the study by Ramachandran [55]. Examining the adjusted data for BMI and WC, the two studies [40,55] that used ITT analyses and LOCF method reported the most conservative outcome results, but this is based on limited data.

**Table 2:** Study outcomes.

Study ID	No. Quality Criteria Met (Max 6)	Effectiveness BMI/z-Score * ↓↑↔?	Effectiveness WC * ↓↑↔?	Effectiveness WT *↓↑↔?	Summary Effectiveness ↓↑↔↕?
**RCTs (children)**					
Singhal 2010 [59]	5	↔	↑	↔	↕
Nidhi 2012 [53]	4	↔	↔	NR	↔
Johnston 2013 [46]	5	↔	NR	↑	↕
**CCTs (children)**					
Almas 2013 [35]	3	↓	NR	NR	↓
Adab 2014 [62]	4	↑ adjusted ↔unadjusted	↔	NR	↕
**BA (children)**					
Balagopal 2008 [38]	4	↓	↑	NR	↕
Kameswararao 2009 [47]	3	?	NR	NR	?
Madsen 2009 [50]	1	↑	NR	NR	↑
**RCTs (adults)**					
Ramachandran 2006 [54]	6	↔	↔	↔	↔
Bellary 2008 [40]	5	↓	↔	↓	↕
Admiraal 2013 [34]	4	↔	↔	↔	↔
Andersen 2013 [36]	6	↑	↑	↑	↑
Ramachandran 2013 [55]	5	↔	↔	NR	↔
Telle-Hjellset 2013 [60]	6	↔	↔	↔	↔
Bhopal 2014 [41]	5	↑ adjusted ↔unadjusted	↑adjusted ↔ unadjusted	↑adjusted ↔ unadjusted	↑adjusted ↔ unadjusted
**BA (adults)**					
Ghosh 2006 [44]	3	↑	NR	NR	↑
Mathews 2007 [51]	2	↑	↔	↑	↕
Rush 2007 [56]	3	NR	↑M ↔F	↑M ↔F	↕
Backes 2008 [37]	2	↑	NR	↑	↑
Balagopal 2008 [38]	4	↓	↑	NR	↕
Dixon 2008 [43]	5	↑	↔	↔	↕
Kousar 2008 [49]	3	↑	NR	NR	↑
Misra 2008 [52]	3	↔	↑	NR	↕
Prabhakaran 2009 [61]	2	NR	↑	↑	↑
Sharma 2009 [58]	0	↑	↑	↑	↑
Balagopal 2012 [39]	4	↑	↑	NR	↑
Chander 2013 [42]	0	↑	NR	NR	↑
Khaskheli 2013 [48]	1	↑	NR	NR	↑
Shailaja 2011 [57]	2	↑	↑	?	↑
Gulati 2014 [45]	4	NR	?	?	?

*↓: intervention not effective; ↑: intervention effective; ↔ intervention equally effective/not effective; **↕** mixed results by outcome or gender; ?: unable to assess effectiveness; note: for CCT/RCTs effectiveness assessed using between group differences, for BA studies effectiveness assessed using within group differences from baseline to last follow-up.

### 3.3. Diet and Physical Activity Interventions in Children

There was three RCTs [46,53,59] two CCTs [35,62] and three BAs [38,47,50] of school-based D and PA interventions in children aged between six and sixteen years. None of the studies reported obesity outcomes by SES.

#### Controlled Clinical Trials/Randomised Controlled Trials

Three studies showed mixed results for outcomes. Two-year follow-up of a 1-year school and family-based D and PA intervention (BEACHeS) [62] in 6-year old South Asian children in the UK, demonstrated significant differences in favour of the intervention for adjusted zBMI and risk of obesity, but not other anthropometric measures (or unadjusted zBMI). One US-based RCT [46] of D and PA provided additional data for a small South Asian subgroup of overweight/obese 8 year old children. At 24-months, there was a significant difference in favour of the intervention for WT; however there was no significant difference between intervention and control for BMI and zBMI. An RCT [59] of D and PA for 6-months in 16-year old school-children in India showed improvement in WC but no difference in BMI between groups. 

One RCT [53] showed no significant difference in BMI or WC between groups of 16-year old school girls in India with polycystic ovary syndrome following a 3-month yoga intervention. Another study showed significant improvement in the control group compared to the intervention group for WT following a 5-month PA intervention in 10-year old school girls in Pakistan; the control group lost more weight than the intervention group [35]. 

**Figure 2:**
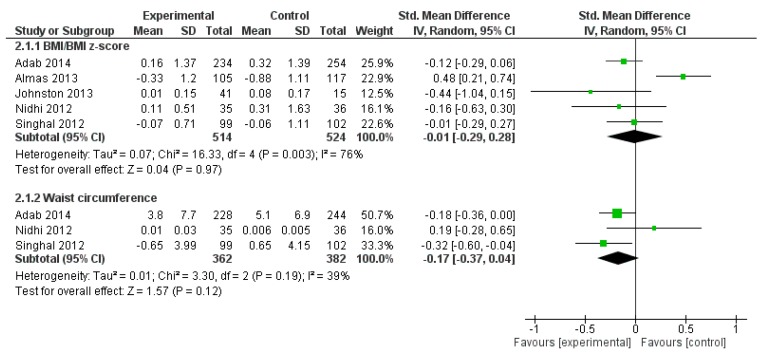
Meta-analyses of mean change in obesity outcomes from baseline to post-intervention for South Asian Children.

All five RCTs/CCTs were suitable for inclusion in meta-analyses of obesity outcomes (Figure 2). There was insufficient data to perform a meta-analysis of WT.

There was no significant between group difference in BMI/zBMI or WC for children (SMD −0.01, 95% CI −0.29 to 0.28; SMD −0.17, 95% CI −0.37 to 0.04, respectively); BMI/zBMI was associated with significant heterogeneity. Sensitivity analysis of BMI/zBMI (Figure S2) was performed by excluding an outlying study [35] which removed heterogeneity; SMD remained non-significant (−0.11, 95% CI −0.25 to 0.03). One study (BEACHeS) [62] also reported mean difference in zBMI and WC adjusted for baseline differences, age, sex and ethnicity; the adjusted difference was significant for zBMI (adjusted MD −0.15, 95% CI −0.27 to −0.03) but not WC (adjusted MD −0.86 95% CI −1.87 to 0.15).

Of the five RCTs/CCTs reporting unadjusted change in BMI/zBMI in children, two were based in non-Asian countries and three were based in South Asia [35,53,59]. Sensitivity analysis by country (South Asian *vs*. non South Asian) showed no difference in treatment effect for BMI/zBMI for children; there was insufficient data to assess this for WC or WT.

#### Before-and-After Studies

The results for the three BA studies were heterogeneous. One community-based study [50] showed significant improvement in zBMI following 8-months PA in a subgroup of 10-year old South Asian children in the USA. Another community-based study [38] of D and PA in 14-year old children in India, showed mixed effectiveness by outcome; BMI increased and WC decreased after 7-months. In another study, two of 59 Indian school-children were no longer obese after a 6-month D and PA intervention; the effectiveness of this intervention is unclear [47].

### 3.4. Diet and Physical Activity Interventions in Adults

**Figure 3:**
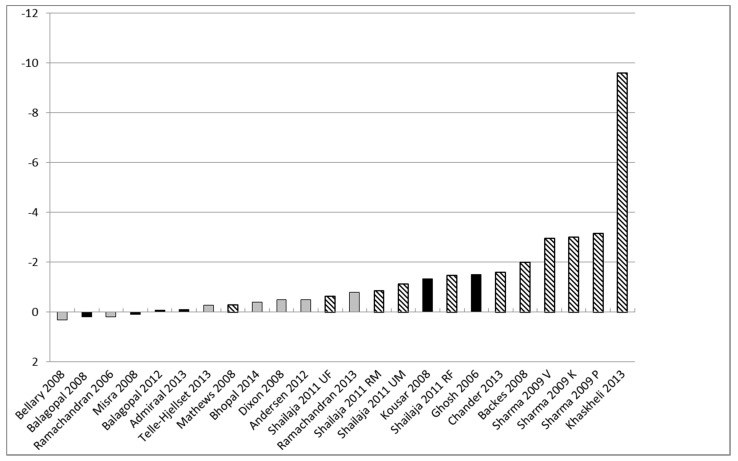
Mean change in BMI for adults from baseline to follow-up for intervention groups. grey = high quality (quality score 5–6); black = medium quality (quality score 3–4); pattern = low quality (quality score 0–2).

There were seven RCTs [34,36,40,41,54,55,60] and 15 BAs [37,38,39,42,43,44,45,48,49,51,52,56,57,58,61] of D and PA interventions in adults. BMI was the most commonly reported obesity outcome in adults; Figure 3 shows change in BMI from baseline to follow-up in intervention arms only (24 arms from 19 studies; three studies did not report BMI). The quality of these 19 studies was assessed as: seven “high”, six “medium” and six “low”. The data suggests, all other things being equal, that lower quality studies were associated with larger changes in BMI. Mean change in BMI from baseline to follow-up ranged from −0.3 kgs to −9.6 kgs for low quality studies, 0.2 kgs to −1.5 kgs for medium quality studies and 0.31 kgs to −0.8 kgs for high quality studies. 

We acknowledge that other factors, in addition to methodological quality, may have contributed to these results. Only two studies reported obesity outcomes by SES; D and PA interventions were equally effective in reducing BMI from baseline to follow-up in Asian adults in India [39] and Pakistani females living in Norway [60], regardless of level of SES. 

#### Randomised Controlled Trials

There were seven RCTs of D and PA interventions in adults. Two high quality RCTs [41,65] reported significant improvements in obesity outcomes. Structured group exercise sessions, twice weekly for five months in Norwegian-speaking Pakistani men, significantly lowered BMI, WC and WT (the control group increased BMI and WC) [65]. Home-visits by dietitians advising Indian and Pakistani adults at high risk of type 2 diabetes in the UK, and their families, on an energy-deficit diet and at least 30 min daily brisk walking for three years, significantly lowered BMI, WC and WT when adjusted for baseline differences between groups, ethnicity and location [41].

One RCT [40] showed mixed effectiveness by outcomes; at 2-year follow-up BMI had increased in the intervention group compared with control, however there was no difference in WC between the groups. Four RCTs [34,54,55,60] showed no significant difference between intervention and control groups at follow-up for BMI, WC or WT.

**Figure 4:**
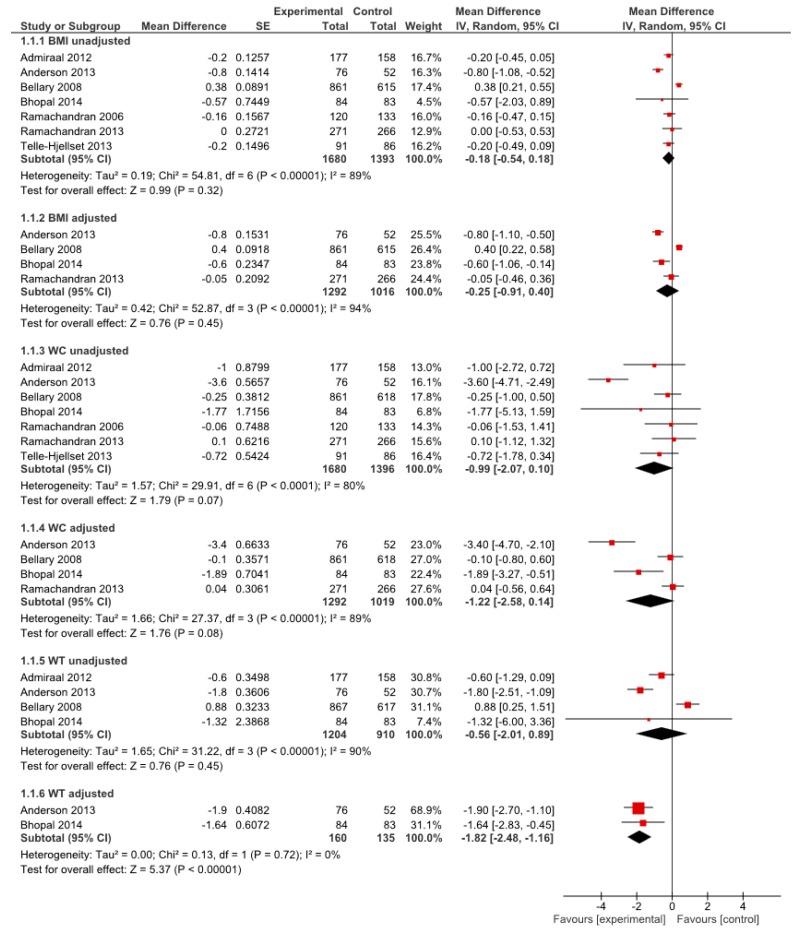
Meta-analyses of mean change in obesity outcomes from baseline to post-intervention for South Asian Adults.

All seven RCTs were suitable for inclusion in meta-analyses of obesity outcomes (Figure 4). There were too few studies included within the meta-analyses to use a funnel plot to explore publication bias [74]. Meta-analyses of unadjusted and adjusted mean differences for BMI and WC demonstrated no significant improvement in favour of intervention (MD −0.18, 95% CI −0.54 to 0.18 unadjusted, MD −0.25 95% CI −0.91 to 0.40 adjusted; MD −0.99, 95% CI −2.07 to 0.10 unadjusted, MD −0.56, 95% CI −2.01 to 0.89 adjusted, respectively) and all analyses were associated with significant heterogeneity. 

Meta-analysis of unadjusted mean differences for WT demonstrated no significant improvement in favour of intervention (MD −0.56, 95% CI −2.01 to −0.89), however sensitivity analysis (Figure S3) was performed excluding an outlying study [40] which removed heterogeneity and the unadjusted mean difference in WT became significant (MD −1.20, 95% CI −2.23 to −0.17). 

The outlying study [40] was in a group of type 2 diabetics and the intervention was unique in that it included an enhanced care package to achieve targets for blood pressure, lipids, and glycaemic control which in some cases included drug medication; some of the drugs prescribed can lead to weight gain. In support of the evidence from the sensitivity analysis of the unadjusted weight data, the adjusted weight data from two studies demonstrated significant improvement in WT (MD −1.82, 95% CI −2.48 to −1.16).

Sensitivity analysis by country (South Asian *vs*. non South Asian) showed no difference in treatment effect for BMI and WC for adults; there was insufficient data to assess this for WT. Of the seven RCTs reporting unadjusted change in BMI in adults, five were based in non-Asian countries and two were based in South Asia [54,55]; excluding or including these two studies had no effect on the result. Excluding the two South Asian studies from the meta-analysis of unadjusted change in WC produced a significant effect in favour of the intervention. However when heterogeneity is eliminated by excluding an outlying study [36] the effect becomes nonsignificant. In summary, the BMI and WC results do not differ depending on country and there is insufficient evidence to assess this for WT.

Two RCTs included men only [36,55], one RCT included women only [60] and four RCTs included both men and women but did not report on potential sex differences in results for BMI/WC/WT. Sensitivity analysis of BMI data in the meta-analysis by gender had no impact on the effect. 

#### Before-and-After Studies

There were 15 adult BA studies measuring change from baseline to follow-up. Nine studies [37,39,42,44,48,49,57,58,61] showed significant improvements in obesity outcomes and five studies showed mixed effectiveness by outcomes [38,43,51,52] or by gender [56]. In one study the statistical significance of change in obesity outcomes was unclear; participants in a dietary control group reduced WC by 3.5 cm and reduced WT by 1.4 kgs [45]. 

### 3.5. Analysis by Type of Intervention and Length of Follow-Up

There was no difference in treatment effect of prevention studies (no limit on BMI in study inclusion) compared with treatment studies (minimum BMI stated as part of study inclusion criteria) from the RCTs/CCTs data. However, using intervention arm data from studies of all designs, the amount of weight loss in intervention groups where participants had a mean baseline BMI above 30 kgs/m^2^ tended to be greater.

Across all study designs there did not appear to be any pattern between effectiveness and length of follow-up. Within the meta-analyses there were too few studies to formally assess whether length of follow-up moderated effects of outcomes. For adults, the largest absolute improvements in outcomes were found in an RCT [36] with the shortest follow-up time of 5 months however there were significant improvements in outcomes in another RCT with 36 month follow-up [41].

Following a valuable suggestion by one of the Journal reviewers we undertook a single meta-analysis and meta-regression (Table S4). The pooled effect from a random effect model was significantly different from zero, but with substantial evidence of heterogeneity between the studies (86%). Meta-regression showed that 92% of the variance from the single meta-analysis could be explained by group (adult/child), outcome (BMI, WC, WT) or study identifier (author).

### 3.6. Economic Outcomes

Two RCTs undertook economic evaluation; the incremental cost-effectiveness ratio of a nurse-led enhanced diabetes care intervention was not cost effective at £28,933 per QALY gained [40]. The additional 3-year cost of a “lifestyle” intervention for South Asian adults at high risk of type 2 diabetes, in terms of health-service costs and indirect costs to participants, was £1126 per participant [41]. 

## 4. Discussion 

The review identified 29 studies which evaluated D and PA interventions in South Asian populations. Only five of these 29 studies were RCTs/CCTs in children; all five were school-based prevention interventions (two in India, one each in Pakistan, UK and USA) and age at baseline ranged from six to 16. Ten of the adult interventions were targeted at adults with various obesity-related co-morbidities and not aimed exclusively at weight loss/weight gain prevention but at reducing risk factors. 

Only one of the eight meta-analyses showed a significant effect; this was for adjusted weight in adults and contained only two studies. Sensitivity analysis (to remove heterogeneity) of the *unadjusted* weight in adults (removing one of three studies) produced a significant effect. Although we pre-specified that heterogeneity would be explored through sensitivity analyses we did not specify which study characteristics would be examined. We highlight that the outlying study intervention [40] differed from the other included interventions and suggest this may be a potential reason for the difference in effect. However, caution should be used against reliance on results obtained from sensitivity analysis particularly with such a small number of pooled studies. The majority of BA studies showed significant improvements in obesity outcomes from baseline to follow-up, and most of the intervention groups of the controlled trials also showed significant improvements in obesity outcomes from baseline to follow-up (despite, in many cases, no significant difference compared to control). Of 19 adult studies that reported BMI for 24 intervention groups, only four groups did not reduce BMI from baseline to follow-up. The absolute reduction in BMI from baseline to follow-up in the intervention groups in BA studies was generally larger than that in the intervention groups of controlled trials. This pattern is reflected in the relationship between quality and BMI changes. Intervention group improvements in BMI were, as expected, smaller in studies of higher methodological quality, and higher in adults with a greater BMI at baseline.

Baseline BMI across the RCTs ranged between 26 to 31 kg/m^2^ whereas baseline BMI across the BA studies had a wider range of 21 to 36 kg/m^2^ and this may have moderated the degree of change in BMI in studies from these two designs. Two community-based interventions in India [38,39] which had the lowest adult baseline BMI of 21 kg/m^2^ were associated with a relatively small change in BMI at follow-up (+0.2 kg/m^2^ and −0.09 kg/m^2^). A study set in a private clinic in Pakistan [48] had the highest adult baseline BMI of 36 kg/m^2^ and was associated with the greatest change in BMI at follow-up (−9.6 kg/m^2^). 

One of the striking findings of this review was the lack of data on cost effectiveness of interventions to prevent or treat obesity in South Asian children or adults. 

Eleven of the fifteen adult BA studies did not report on all three obesity outcomes whereas six of the seven adult controlled trials reported all three outcomes. Summary of effectiveness was based on all reported outcomes and so this may partially explain some of the discrepancy between improvements in outcomes in BA studies but not in controlled trials. In addition, most of the BA studies were short-term (less than 12 months) whereas five of the seven adult RCTs were longer-term.

Average change in BMI from baseline to follow-up from high quality studies of adults (intervention groups only) ranged from 0.31 to −0.8 kg/m^2^. We found no evidence that these interventions were more or less effective according to whether the intervention was set in South Asia or not, or by SES. 

When evaluating process characteristics of effective or promising trials there appears to be some interesting evidence around school-based PA. BEACHeS [62] demonstrated significant improvement in BMI when adjusted for baseline differences; study authors reported that whilst certain types of PA were culturally unacceptable for girls, increasing opportunities within school for PA was acceptable. Family cooking skills workshops, signposting of local leisure facilities and attending a day event at a local football club, were feasible and acceptable, however promoting walking groups was neither feasible nor acceptable.

Following a 5-month school-based PA intervention in Pakistan with girls aged 9–11 years, the control group lost more weight than the intervention group [35]. Study authors reported that 40 girls were not allowed to participate as it was thought culturally inappropriate by their parents, however, regular sessions to discuss acceptability of the PA intervention helped to retain participants. Another school-based D and PA intervention [59] that showed significant improvement in WC but not BMI and WT between intervention and control at 6-months used peer educators and student volunteers to aid sustainability of the intervention.

Two promising adult studies [36,41] were high quality RCTs with low attrition. There are various potential explanations postulated by the authors of the UK PODOSA trial [41] for its improvements in obesity outcomes when adjusted for baseline differences in South Asians of Indian or Pakistani origin. Unlike any of the other included adult studies, PODOSA was family-based and home-based; the support of the family cook was mandatory for enrolment. In addition the culturally adapted D and PA intervention was moderately intense and was the longest of the included RCTs (three years). It was delivered by dietitians (15 visits over three years), who played a central role in the study. The study was targeted at South Asians at high risk of type 2 diabetes, whose baseline BMI was 31 kg/m^2^.

The other remaining effective adult RCT appears quite different from PODOSA; it was set in Norway and recruited only men of Pakistani origin [36]. This was the only included study that used PA alone in South Asian men set in a non-Asian country. The men were relatively young adults (mean age 36), physically inactive, and approximately half the men had metabolic syndrome; mean baseline BMI was 27 kgs/m^2^. The intervention focused on PA alone and was five months duration. The study authors report that the intervention was developed in collaboration with the Pakistani community, used a social cognitive theory framework, and consisted of structured supervised group exercises for 60 minutes twice per week, group lectures, individual counselling and telephone follow-up. This study showed significant improvement compared to control for all obesity outcomes and in both unadjusted and adjusted data.

### 4.1. Strengths and Limitations of this Review

This is the first review to the author’s knowledge, that synthesises the evidence for D and PA interventions conducted in any country, any setting and across all ages of South Asian populations. A balance of studies conducted in Asian and non-Asian countries were identified, with findings relevant for South Asians living in or outside South Asia. This is an important strength of this review; all previous reviews either focus on interventions set in South Asia or outside of South Asia [23]. 

The inclusion criteria for this review was kept deliberately wide to capture as much evidence as possible on effectiveness, however this also means that the quality of evidence varied considerably. Despite a comprehensive search, this review identified only three RCTs in South Asian children and seven RCTs in South Asian adults. Despite a comprehensive search it is possible that studies which undertook subgroup analysis of anthropometric outcomes by South Asian ethnicity but did not report this in the abstract were missed. Although we describe the characteristics of effective or promising interventions as identified from the studies we included in this review in order to aid understanding of what components of interventions might “work”, this is not intended as a thorough review of the literature. Many qualitative and quantitative studies of a variety of designs have been conducted, including process evaluations of interventions, which provide a rich source of information on the characteristics of, and approaches to, successful public health interventions targeted at South Asian populations. 

Twenty-seven of the 29 studies were based exclusively in South Asian populations. There were no studies of Sri Lankans. The findings of ongoing RCTs [34,73,75,76] will make an important contribution to the evidence base.

Due to inclusion of different study designs, in some studies effectiveness was assessed using between group differences, in other studies effectiveness was assessed using within group differences from baseline to last follow-up. This meant that in RCTs, where an outcome was significantly reduced from baseline to follow-up in the intervention group but not significantly different between groups at follow-up, effectiveness was classified as equally effective (↔). In single-group BA studies, where an outcome was significantly reduced from baseline to follow-up the outcome was classified as effective (↑). This is important to bear in mind when evaluating the results across different study designs. 

We attempted to synthesis as much available evidence as possible and so we included both adjusted and unadjusted data. There is no consensus about whether or how to synthesise adjusted and unadjusted findings in a research synthesis; within the adult meta-analyses we present both unadjusted and adjusted results. Difficulty interpreting findings arises, however, when adjusted and unadjusted findings do not support the same conclusion as was the case with individual study data (significant adjusted, non-significant unadjusted) for the BEACHeS [62] and PODOSA studies [41]. However, meta-analyses of adjusted and unadjusted data did not show discrepancy between these types of data for overall effect measures. 

We looked in detail at how individual studies accounted for participants who were lost to follow-up; most studies performed analysis on completers only, although two studies [40,55] carried out ITT analysis. Completer analysis could potentially overestimate any effect of the intervention due to attrition bias (assuming participants who dropped out lost less weight compared with those who completed the study). However, due to a number of factors, include the relatively few controlled studies that were included in the meta-analyses, and heterogeneity of results, we did not make any adjustment for how studies accounted for attrition in our analysis (although we did include some weighting for attrition in the quality assessment). As a result, we acknowledge that the results presented in this review may be prone to attrition bias.

In summary, the main limitations to the data relate to having a relatively small number of controlled trials that were suitable for meta-analyses. Evidence from BA studies should be regarded with some caution; improvements in BMI in intervention participants from baseline to follow-up could be confounded by a number of factors. Indeed, this limitation is supported by the evidence that higher quality studies were associated with smaller improvements in BMI and, ultimately, no significant difference was found between intervention and control groups for BMI in meta-analyses, even when BMI was adjusted for baseline differences. 

### 4.2. Recommendations for Research

We have described the types of interventions that show promise but we do not know which specific intervention components are effective. We recommend more obesity interventions targeting South Asian populations are conducted, particularly those targeting pre-school children and their families. These studies should report (1) how interventions are culturally adapted; (2) the types of behaviour change techniques and theories that are used to underpin interventions; (3) anthropometric outcomes by measures of SES; and (4) implementation and running costs. These recommendations would enable reviewers to assess how behaviour change techniques and theories moderate effectiveness, to assess the equity impacts of interventions, and to examine explanations for heterogeneity between interventions. The development of effective interventions may well require a great deal of qualitative and quantitative research on knowledge, attitudes, behaviours, and perceptions. More research is needed into the differential effects of lifestyle interventions for South Asians compared with other ethnicities. The general approach so far has been to culturally adapt existing interventions. However, there may well be a need to develop new interventions from first principles.

## 5. Conclusions

This review is unique in combining the evidence from interventions carried out in South Asian populations living in both South Asia and in other countries. Twenty-nine studies were included which evaluated diet and physical activity interventions in South Asian children and adults. Meta-analysis of a limited number of controlled trials found an unclear picture of the effects of interventions on BMI for South Asian children. Meta-analyses of a limited number of controlled trials showed a significant improvement in adult weight but no significant differences in BMI and WC. The majority of intervention groups from all types of study designs included in this review showed significant improvements in BMI from baseline to follow-up. Average change in BMI in intervention groups, from baseline to follow-up, in high quality studies of adults ranged from 0.31 to −0.8 kg/m^2^. It is important to note that these results should be interpreted with some degree of caution given the quality of evidence available for review. We found no evidence that these interventions were more or less effective according to whether the intervention was set in South Asia or not, or by SES. One high quality RCT in South Asian children found that a school-based physical activity intervention that was delivered within the normal school day which was culturally sensitive, was effective. There is also evidence of culturally appropriate approaches to, and characteristics of, effective interventions in adults which we believe could be transferred and used to develop effective interventions in children.

## Supplementary Files

Supplementary File 1
