# Supplementary material for: Diet and Physical Activity Interventions to Prevent or Treat Obesity in South Asian Children and Adults: A Systematic Review and Meta-Analysis
Source: Int J Environ Res Public Health. 2015 Jan 9;12(1):566–94. doi: 10.3390/ijerph120100566 (PMC4306880; doi:10.3390/ijerph120100566)
Supplement: Supplementary File 1 [file ijerph-12-00566-s001.pdf]

Supplementary Information

Diet and Physical Activity Interventions to Prevent or Treat Obesity in South Asian Children and Adults: A Systematic Review and Meta-Analysis

---

Figure S1. Electronic Search Strategies

Ran 23/01/14 in Medline 1946 to January week 3, 2014
1. exp OBESITY/
2. exp Weight Gain/
3. exp Weight Loss/
4. "obes*".ti,ab.
5. (weight gain or weight loss).ti,ab.
6. (overweight or over weight).ti,ab.
7. "weight change*".ti,ab.
8. ((bmi or body mass index) adj2 (gain or loss or change)).ti,ab.
9. or/1-8
10. activ$.ti,ab.
11. exercis$.ti,ab.
12. nutrition$.ti,ab.
13. diet$.ti,ab.
14. eat$.ti,ab.
15. food$.ti,ab.
16. or/10-15
17. exp asian continental ancestry group/
18. islam/
19. hinduism/
20. pakistan$.ti,ab.
21. india$.ti,ab.
22. bangladesh$.ti,ab.
23. south asia$.ti,ab.
24. or/17-23
25. 9 and 16
26. 25 and 24
27. limit 26 to (abstracts and humans and yr="2006 -Current")

Ran 23/01/14 in Embase 1980 to 2014 week 3
1. exp OBESITY/
2. exp Weight Gain/
3. exp Weight Reduction/
4. "obes*".ti,ab.
5. (weight gain or weight loss).ti,ab.
6. (overweight or over weight).ti,ab.
7. "weight change*".ti,ab.
8. ((bmi or body mass index) adj2 (gain or loss or change)).ti,ab.
9. or/1-8
10. activ$.ti,ab.
11. exercis$.ti,ab.
12. nutrition$.ti,ab.

13. diet$.ti,ab.
14. eat$.ti,ab.
15. food$.ti,ab.
16. or/10-15
17. exp asian continental ancestry group/
18. religion/
19. pakistan$.ti,ab.
20. india$.ti,ab.
21. bangladesh$.ti,ab.
22. south asia$.ti,ab.
23. or/17-22
24. 9 and 16
25. 24 and 23
26. limit 25 to (abstracts and human and yr="2006 -Current")

Ran 22/01/14 in CCTR
#1 MeSH descriptor: [Obesity] explode all trees
#2 MeSH descriptor: [Body Weight Changes] explode all trees
#3 obes*
#4 ("weight gain" or "weight loss")
#5 (overweight or "over weight")
#6 (weight next change*)
#7 ((bmi or "body mass index") near/2 (gain or loss or change*))
#8 #1 or #2 or #3 or #4 or #5 or #6 or #7
#9 activ*
#10 exercis*
#11 nutrition*
#12 diet*
#13 eat*
#14 food*
#15 #9 or #10 or #11 or #12 or #13 or #14
#16 MeSH descriptor: [Asian Continental Ancestry Group] explode all trees
#17 MeSH descriptor: [Islam] this term only
#18 india*
#19 Bangladesh*
#20 South next asia*
#21 #16 or #17 or #18 or #19 or #20
#22 #8 and #15
#23 #21 and #22
#24 #21 and #22 from 2006 to 2014, in Trials

Ran 23/01/14 in ASSIA: ab(Asian) AND ab(weight OR exercise OR diet) From 01 January 2006 to 31 January 2014.

Ran 23/01/14 in SSCI: TOPIC: *(exercise or diet or weight) AND* TITLE: *(asian)*, Timespan=2006-2014. Indexes=SSCI.

Table S1. Quality assessment.

STUDY ID	Representativeness *	Randomisation **	Comparability ***	Credibility +	Attrition ++	Attributability +++
Adab 2014 [1]	No	No	Yes	Yes	Yes	Yes
Admiraal 2013 [2]	No	Yes	Yes	Yes	No	Yes
Almas 2013 [3]	No	No	Unclear	Yes	Yes	Yes
Andersen 2013 [4]	Yes	Yes	Yes	Yes	Yes	Yes
Backes 2008 [5]	No	N/A	N/A	Unclear	Yes	Yes
Balagopal 2008 [6]	Yes	N/A	N/A	Yes	Yes	Yes
Balagopal 2012 [7]	Yes	N/A	N/A	Yes	Yes	Yes
Bellary 2008 [8]	No	Yes	Yes	Yes	Yes	Yes
Bhopal 2014 [9]	Unclear	Yes	Yes	Yes	Yes	Yes
Chander 2013 [10]	No	N/A	N/A	Unclear	No	Unclear
Dixon 2008 [11]	No	N/A#	N/A#	Yes	Yes	Yes
Ghosh 2006 [12]	No	N/A	N/A	Yes	Yes	Yes
Gulati 2014 [13]	No	N/A#	N/A#	Yes	Yes	Yes
Johnston 2013 [14]	No	Yes	Yes	Yes	Yes	Yes
Kameswararao 2009 [15]	Unclear	N/A#	N/A#	Yes	Yes	Yes
Khaskheli 2013 [16]	No	N/A	N/A	Unclear	Yes	Unclear
Kousar 2008 [17]	No	N/A	N/A	Yes	Yes	Yes

Table S1. *Cont.*

STUDY ID	Representativeness *	Randomisation **	Comparability ***	Credibility +	Attrition ++	Attributability +++
Madsen 2009 [18]	Unclear	N/A	N/A	Yes	No	Unclear
Mathews 2007 [19]	Yes	N/A	N/A	Yes	No	Unclear
Misra 2008 [20]	No	N/A	N/A	Yes	Yes	Yes
Nidhi 2012 [21]	No	Yes	No	Yes	Yes	Yes
Prabhakaran 2009 [22]	Yes	No	Unclear	Yes	No	Unclear
Ramanchandran 2006 [23]	Yes	Yes	Yes	Yes	Yes	Yes
Ramanchandran 2013 [24]	No	Yes	Yes	Yes	Yes	Yes
Rush 2007[25]	Yes	N/A	N/A	Yes	Unclear	Yes
Shailaja 2011 [26]	Unclear	No	Yes	Yes	No	Unclear
Sharma 2009 [27]	No	No	No	Unclear	N/A	Unclear
Singhal 2010 [28]	Yes	Yes	Unclear	Yes	Yes	Yes
Telle-Hjellset 2013 [29]	Yes	Yes	Yes	Yes	Yes	Yes

only the dietary control arm was eligible for inclusion in this review; * Representativeness: Were the study samples randomly recruited from the study population with a response rate of at least 60% or were they otherwise shown to be representative of the study population? ** Randomisation: Were participants, groups or areas randomly allocated to receive the intervention or control condition? Not applicable (N/A) for all study designs except controlled trials; ***Comparability: Were the baseline characteristics of the comparison groups comparable or if there were important differences in potential confounders were these appropriately adjusted for in the analysis? If there is no comparison group this criterion cannot be met. Not applicable (N/A) for all study designs except controlled trials; + Credibility of data collection instruments: Were data collection tools shown to be credible, e.g., shown to be valid and reliable in published research or in a pilot study, or taken from a published national survey, or recognized as an acceptable measure; ++ Attrition Rate: Were outcomes studied in a panel of respondents with an attrition rate of less than 30%? +++ Attributability to intervention: Is it reasonably likely that the observed effects were attributable to the intervention under investigation? This criterion cannot be met if there is evidence of contamination of a control group in a controlled study. Equally, in all types of study, if there is evidence of a concurrent intervention that could also have explained the observed effects and was not adjusted for in analysis, this criterion cannot be met; randomisation and comparability are not applicable (N/A) for all study designs except controlled trials.

Table S2. Study characteristics.

Study ID	Methods	Participants	Intervention	Outcomes	Implementation
Adab 2014 [1] Birmingham healthy Eating, Active lifestyle for Children Study (BEACHeS)	**Design:** Cluster CCT<br>**Aim:** To assess feasibility and acceptability of the intervention and obtain data to inform sample size for a definitive trial<br>**Duration:** 2 years (1 year intervention plus another year follow-up)<br>**Power:** not powered to examine outcomes<br>**ITT:** No	**Age (years):** I: 6.53; C: 6.44<br>**Sex (M/F):** I: 53.5 C: 49.8% M<br>**South Asian:** 85.9%<br>**Baseline BMI z-score**: I: −0.03 (1.37); C: 0.08 (1.39)<br>**Health status:** 1 in 5 were overweight<br>**Number:**574<br>**Setting:** Schools<br>**Country:** Birmingham, UK	**Intervention:** school-based and family-based activities, targeting D and PA (modified and refined throughout)<br>**Theoretical framework**: theoretical and modelling phases of the MRC framework to develop intervention, ANGELO framework, 'Theories of Change' approach<br>**Control:** non-intervention	**Reported:** Bioimpedance<br>BMI z-score<br>Skinfolds<br>WC<br>Weight status<br>**Subgroup analysis by SES:** No, reports free school meal eligibility and Townsend Deprivation Scores, >90% were from the most deprived areas	**Funding of intervention:** funded by the National Prevention Research Initiative (NPRI), Grant No. G0501292; funding partners include British Heart Foundation; Cancer Research UK; Department of Health; Diabetes UK; Economic and Social Research Council; Medical Research Council; Research and Development Office for Northern Ireland Health and Social Services; Chief Scientist Office, Scottish Executive Health Department; Welsh Assembly Government, World Cancer Research Fund<br>**Funding of evaluation:** NR<br>**Who delivered:** Trained school staff (teachers, teaching assistants or lunch time assistants); Aston Villa Football Club Community programme Staff; Birmingham Community NHS Trust dietetics staff; research staff<br>**Other resources:** most intervention components were adapted from existing services and facilities commissioned by the local NHS. The resources for training teachers to deliver structured PA sessions are available commercially to schools<br>**Economic evaluation:** NR<br>**Process evaluation:** Acceptability, feasibility

Table S2. *Cont.*

Study ID	Methods	Participants	Intervention	Outcomes	Implementation
Admiraal 2013 [2]	**Design:** RCT<br>**Aim:** To prevent diabetes<br>**Duration:** 1 year<br>**Power:** Assuming an alpha of 5%, study had a power of 81% to demonstrate a minimum difference in weight reduction of 1.25 kg<br>**ITT:** No	**Age (years):** 44.9<br>**Sex (M/F):** 50.7% M<br>**South Asian:** 100% Hindustani Surinamese<br>**Baseline BMI**: I: 28.1 (3.9); C: 27.2 (3.8)<br>**Health status:** At risk of diabetes<br>**Number:** 536<br>**Setting:** General Practice<br>**Country:** The Hague, The Netherlands	**Intervention:** culturally adapted, individual diet and exercise counselling (6 to 8 sessions in first 6 months, then 2 booster sessions next 6 months, 1 family session, 2 cooking classes, 20-week supervised PA programme<br>**Theoretical framework**: Motivational interviewing<br>**Control:** lifestyle advice	**Reported:**<br>% fat mass<br>BP<br>BMI<br>Glucose<br>Hip circ<br>Lipids<br>Waist circ<br>Weight<br>**Subgroup analysis by SES:** No	**Funding of intervention:** Dutch organisation for Health Research and Development (ZonMW, project number 120620023)<br>**Funding of evaluation:** NR<br>**Who delivered:** dieticians, PA coaches, interviewer, research staff<br>**Other resources:** NR<br>**Economic evaluation:** NR<br>**Process evaluation:** NR
Almas 2013 [3]	**Design:** Cluster CCT<br>**Aim:** To determine feasibility<br>**Duration:** 20-weeks<br>**Power:** No for feasibility, yes for BP, unclear for BMI<br>**ITT:** No	**Age (years):** 10.2 (1.1), 9–11<br>**Sex (M/F):** 100% F<br>**South Asian:** 100%<br>**Baseline BMI z-score:** I: 1.35 (1.39); C: 1.92 (1.82)<br>**Health status:** Overweight otherwise healthy<br>**Number:** 280<br>**Setting:** 4 public sector schools<br>**Country:** Karachi, Pakistan	**Intervention:** 20-week school-based PA programme of 30 min, 4x week, in addition to usual care, no music or dance<br>**Theoretical framework**: psychosocial cognitive theory<br>**Control:** usual care (free play in break times) plus 1x 30 min session on healthy diet and PA (food pyramid)	**Reported:**<br>BP<br>BMI<br>**Subgroup analysis by SES:** No	**Funding of intervention:** Aga Khan University (743-CHS/ERC-07) (Clinical trial ID NCT 00533819)<br>**Funding of evaluation:** NR<br>**Who delivered:** physical trainer<br>**Other resources:** designed by certified sports educationists and paediatrician<br>**Economic evaluation:** No<br>**Process evaluation:** fidelity, acceptability

Table S2. *Cont.*

Study ID	Methods	Participants	Intervention	Outcomes	Implementation
Andersen 2013 [4]	**Design:** RCT<br>**Aim:** To explore effect of PA on MetS<br>**Duration:** 5 months (PA outcomes at 11 months)<br>**Power:** based on PA: with a power of 0.8, a significance level of 0.05 and a presumed drop-out rate of 20 %, a total of 144 participants were needed (not powered to detect changes in MetS)<br>**ITT:** No	**Age (years):** I:35.7; C:39.7<br>**Sex (M/F):** 100% M<br>**South Asian:** 100% Pakistani immigrants (either born in Pakistan or parents born in Pakistan<br>**Baseline BMI:** I: 27.1 (3.2); C: 27.4 (4.2)<br>**Health status:** not physically active; 47%–51% MetS<br>**Number:** 150<br>**Setting:** Community and University<br>**Country:** Oslo, Norway	**Intervention:** structured group exercise sessions 2x week, 2x group lectures, 1x individual counselling session, written material and a phone call<br>**Theoretical framework**: social cognitive theory<br>**Control:** received baseline results and were offered organized exercise, 1x group lecture and written material	**Reported:**<br>BP<br>Glucose<br>Lipids<br>MetS<br>MetS components<br>PA<br>Waist circ<br>**Subgroup analysis by SES:** No	**Funding of intervention:** Norwegian Extra Foundation for Health and Rehabilitation, and Norwegian School of Sport Sciences<br>**Funding of evaluation:** NR<br>**Who delivered:** exercise physiologist<br>**Other resources:** NR<br>**Economic evaluation:** NR<br>**Process evaluation:** adherence
Backes 2008 [5]	**Design:** single-arm before and after study<br>**Aim:** To determine effectiveness on weight loss, insulin sensitivity, and associated cardiovascular disease risk factors<br>**Duration:** 3 month<br>**Power:** N/R<br>**ITT:** No	**Age (years):** 44 (9)<br>**Sex (M/F):** 100% F<br>**South Asian:** 100%<br>**Baseline BMI:** 30.2 (0.8)<br>**Health status:** insulin resistant<br>**Number:** 23<br>**Setting:** General Clinical Research Centre<br>**Country:** Stanford, USA	**Intervention:** 500 kcal deficit diet (40%–45% carb), 1–2 h nutrition education, encouraged not to change level of PA<br>**Theoretical framework**: NR<br>**Control:** None	**Reported:**<br>BP<br>BMI<br>Glucose<br>Lipids<br>Pulse<br>Weight<br>**Subgroup analysis by SES:** No	**Funding of intervention:** General Clinical Research Center, Stanford Medical Center (RR-00070), and a National Institute of Child Health and Human Development K12 Award (5K12HD043452-02)<br>**Funding of evaluation:** NR<br>**Who delivered:** dietician<br>**Other resources:** NR<br>**Economic evaluation:** NR<br>**Process evaluation:** NR

Table S2. *Cont.*

Study ID	Methods	Participants	Intervention	Outcomes	Implementation
Balagopal 2008 [6]	**Design:** single-arm before and after study<br>**Aim:** To prevent/reduce risk of developing diabetes<br>**Duration:** 7 months<br>**Power:** NR<br>**ITT:** No	**Age (years):**<br>**Adults:** 40.3 (15.1)<br>**Youth:** 13.68 (2.0)<br>**Sex (M/F):**<br>**Adult:**41% M<br>**Youth:** 48% M<br>**South Asian:** 100%<br>**Baseline BMI:**<br>**Adults** 20.59 (3.82)<br>**Youth** 16.01 (2.7)<br>**Health status: Adults:** diabetes 5.1%, IFG 13.5%<br>**Youth:** diabetes:0%, IFG 5.1%<br>**Number:** 585 adults, 118 youth<br>**Setting:** 'resource poor' agrarian village<br>**Country:** Tamilnadu, India	**Intervention:** culturally and linguistically appropriate health education messages addressed diet, (increasing fibre, reducing fat, and portion control), PA, and knowledge improvement. Local, low cost dietary resources. Ten face-to-face sessions on a one-on-one basis with health messages tailored for sex, age, and socioeconomic differences. Reinforcement by group events. Individuals with IFG were given additional counselling as needed.<br>**Theoretical framework**: NR<br>**Control:** None	**Reported:**<br>BP<br>BMI<br>Dietary intake<br>Glucose<br>Hip circ<br>Knowledge score<br>Thigh circ<br>Waist circ<br>WHR<br>**Subgroup analysis by SES:** No, despite baseline SES: 51% low SES (income in Rupees <3000 or 75.00/month)	**Funding of intervention:** American Association of Physicians of Indian Origin<br>**Funding of evaluation:** NR<br>**Who delivered: trainers**<br>**Other resources:** trainers were science graduates plus 6-months training<br>**Economic evaluation: Process evaluation:** initial participatory rural analysis enabled the involvement of village leaders, peer educators, and residents in the planning and implementation phases

Table S2. *Cont.*

Study ID	Methods	Participants	Intervention	Outcomes	Implementation
Balagopal 2012 [7]	**Design:** single-arm before and after study<br>**Aim:** To prevent/manage diabetes<br>**Duration:** 6 months<br>**Power:** NR<br>**ITT:** No	**Age (years):** 41.9 (15.9)<br>**Sex (M/F):**<br>**High SES:** 46% M;<br>**Low SES:** 47% M<br>**South Asian:** 100%<br>**Baseline BMI:**<br>High SES: 22.2 (4.7);<br>Low SES: 19.2 (4.1);<br>**Health status:** Overall point prevalence of diabetes, prediabetes, obesity, and hypertension were 7.2%, 19.3%, 16.7%, and 28%, respectively. Significant differences at baseline between high and low SES.<br>**Number:** 1681<br>**Setting:** community, 2 predominantly different target areas "business community" (high SES) reported a below-poverty level of 24% and illiteracy level of 9.7%, whereas the economically stressed agrarian farmworkers (low SES) reported 51% and 50.5%, respectively.<br>**Country:** rural Gujarat, India	**Intervention:** culturally and linguistically appropriate (Gujarati language) health education messages were provided in (*n* = 5) face-to face individual and (*n* = 5) group sessions (demonstrations of model meals and cooking techniques). Local, low cost dietary resources. Physically active participants advised to continue their routine and those engaged in sedentary to light PA advised and regularly motivated to be physically active (e.g., walk, perform household chores, garden, dance/ exercise) for at least 30 min per day.<br>**Theoretical framework**: community-based participatory research<br>**Control:** None	**Reported:**<br>BP<br>BMI<br>CVD knowledge<br>Diabetes knowledge<br>Fruit intake<br>Glucose<br>Moderate/vigorous PA<br>Vegetable intake<br>Waist circ<br>**Subgroup analysis by SES:** Yes	**Funding of intervention:** American Association<br>of Physicians of Indian Origin in collaboration with Texas A&M University<br>and Maharaja Sayajirao University of Baroda<br>**Funding of evaluation:** NR<br>**Who delivered:** project co-ordinator and 16 trained community health workers (CHWs)<br>**Other resources:** All project personnel were formally interviewed. Multidisciplinary team consisting of a dietician, a certified health educator, a public health practitioner,<br>an endocrinologist, a sanitation specialist, a general practitioner, and an internal medicine specialist, provided the 4 weeks training to the CHWs and the project coordinator.<br>CHWs were paid a stipend.<br>**Economic evaluation:** NR<br>**Process evaluation:** key formal and informal community leaders, village elders, local school staff, health professionals, and community members, involved in the planning, implementation, education, and review process

Table S2. *Cont.*

Study ID	Methods	Participants	Intervention	Outcomes	Implementation
Bellary 2008 [8]	**Design:** cluster RCT<br>**Aim:** To investigate the effectiveness of a culturally sensitive, enhanced care package to improve cardiovascular risk profile<br>**Duration:** 2 years<br>**Power:** Yes<br>**ITT:** Yes	**Age (years):** 57.0 (11.9)<br>**Sex (M/F)**: 52% M<br>**South Asian:** 100%<br>**Baseline BMI:** I: 28.5 (4.8); C: 28.6 (4.9)<br>**Health status:** type II diabetes<br>**Number:** 1486<br>**Setting:** 21 inner-city General Practices<br>**Country:** Birmingham and Coventry, UK	**Intervention:** Diet and lifestyle changes, and drug treatment for control of BP, diabetes and lipids. Enhanced care including additional time with practice nurse and support from a link worker (*n* = 5) and diabetes-specialist nurse (*n* = 2). Practice nurses worked with primary-care physicians to implement the protocol and encourage appropriate prescribing, provide face-to-face patient education in clinic setting, and achieve targets for blood pressure, lipid, and glycaemic control. Link workers provided interpretation and additional educational input in local languages (Punjabi, Urdu, and Mirpuri) to patients and attended research clinics. Patients were seen every 2 months by a practice nurse.<br>**Theoretical framework**: NR<br>**Control:** standard care;	**Reported:**<br>BMI<br>BP<br>CHD risk<br>HbA1c<br>Lipids<br>Waist circ<br>**Subgroup analysis by SES:** No	**Funding of intervention:** Laboratories UK, Merck Sharp & Dohme/Schering-Plough, Takeda UK, Roche, Merck Pharma, Daiichi-Sankyo UK, Boehringer Ingelheim, Eli Lilly, NovoNordisk, Bristol-Myers Squibb, Solvay Health Care, and Assurance Medical Society, UK. This trial is registered, number ISRCTN 38297969.<br>**Funding of evaluation:** The sponsors of the study had no role in study design, data collection, data analysis, data interpretation, or writing of the report.<br>**Who delivered:** additional practice nurse time (4 h per practice per week), supported by link workers and a community nurse specialising in diabetes<br>**Other resources:** All link workers had attended a foundation course (equivalent to diploma) in diabetes management and care. Practice nurses were formally trained in diabetes and had 1:1 observed sessions with a diabetes-specialist nurse. Practice nurses had protected time to run a research diabetes clinic in intervention practices.<br>**Economic evaluation:** Yes (ICER)<br>**Process evaluation:** NR

Table S2. *Cont.*

Study ID	Methods	Participants	Intervention	Outcomes	Implementation
			received the same treatment protocols, and practices managed patients with their existing resources.		
Bhopal 2014 [9] Prevention of Diabetes and Obesity in South Asians (PODOSA)	**Design:** cluster RCT<br>**Aim:** To assess a weight control and PA intervention<br>**Duration**: 3 years<br>**Power:** A sample of 150 people assessed at 3 years gave 86% power to detect a mean difference in weight of 2·5 kg between the two groups, assuming an SD of 5 kg with a two-sided 5% significance level.<br>**ITT:** No	**Age (years):** I: 52.8; C:50.2<br>**Sex (M/F):** I: 46% M; C: 45% M<br>**% South Asian:** 100% South Asian descent (Indian, Pakistani)<br>**Baseline BMI;** I: 30.59 (5.02); C: 30.49 (4.60)<br>**Health status:** waist circ 90 cm or greater in men or 80 cm or greater in women, impaired glucose tolerance or impaired fasting glucose<br>**Number randomised:** 156 families<br>**Setting:** home-based<br>**Country:** Glasgow and Edinburgh, UK	**Intervention:** Families had 15 visits from a dietician over 3 years (baseline, monthly for the first 3 months, then every 3 months. The dieticians advised participants and family volunteers on achieving weight loss through a calorie-deficit diet and PA at least 30 min daily brisk walking, using culturally adapted and translated resources. 3-day food diaries and pedometers provided. Family volunteers were asked to follow the advice given and to help the participants to follow it.<br>**Theoretical framework**: NR<br>**Control**: standardised written and verbal advice on healthy eating, diabetes	**Reported:**<br>BMI<br>BP<br>Glucose<br>Hip circ<br>PA<br>Type II diabetes<br>Waist circ<br>Weight<br>WHR<br>**Subgroup analysis by SES:** No	**Funding of intervention:** Medical Research Council (MRC)<br>**Funding of evaluation:** The sponsors of the study had no role in study design, data collection, data analysis, data interpretation, or writing of the report. A representative of the funders, National Prevention Research Initiative (MRC), was a member of the Trial Steering Group.<br>**Who delivered:** dieticians, nurses<br>**Other resources: NR**<br>**Economic evaluation:** Yes, cost analysis<br>**Process evaluation:** NR

Table S2. *Cont.*

Study ID	Methods	Participants	Intervention	Outcomes	Implementation
			prevention, promotion of PA and on accessing other weight control and physical activity services over four visits (baseline, then annually) with a dietician. Advice aimed to halt increasing weight.		
Chander 2013 [10]	**Design:** single-arm before and after study<br>**Aim:** To assess the impact of pharmaceutical care intervention on general cardiovascular risk<br>**Duration:** 10 months<br>**Power:** NR<br>**ITT:** No	**Age (years):** 60 (>45)<br>**Sex (M/F)**: 71% M (*n* = 104)<br>**South Asian:** 100%<br>**Baseline BMI:** 30.64 (3.62)<br>**Health status:** diabetes and/or hypertension<br>**Number:** 157<br>**Setting:** 2 tertiary care hospitals<br>**Country:** Coimbatore, India	**Intervention:** pharmaceutical care intervention consisted of individual follow-ups, medication review, and educative group activities. Counselling on smoking cessation, alcohol reduction, increased compliance, weight reduction, increased exercise, and dietary changes. One visit every 3 months.<br>**Theoretical framework**: NR<br>**Control:** None	**Reported:**<br>10-year risk of heart attack (Framingham score)<br>BP<br>BMI<br>Glucose<br>Lipids<br>**Subgroup analysis by SES:** No	**Funding of intervention:** NR<br>**Funding of evaluation:** NR<br>**Who delivered:** pharmacist<br>**Other resources:** NR<br>**Economic evaluation:** NR<br>**Process evaluation:** NR

Table S2. *Cont.*

Study ID	Methods	Participants	Intervention	Outcomes	Implementation
Dixon 2008 [11]	**Design:** non-blind RCT treated as single-arm before-after study for this review<br>**Aim:** To compare the effect of Orlistat on serum endotoxin lipopolysaccharide and adipocytokines (independent of weight loss)<br>**Duration:** 1 year<br>**Power:** not powered to detect a reduction in progression from IGT to diabetes<br>**ITT:** No	**Age (years):** >25<br>**Sex (M/F):** 8M/7F<br>**South Asian:** 100%<br>**Baseline BMI:** 27.3 (3.1)<br>**Health status:** IGT<br>**Number:** 40<br>**Setting:** Hospital<br>**Country:** Birmingham, UK	Before randomisation all participants received individual dietary advice on healthy eating patterns, portion sizes, 600 kcal⁄day deficit diet with fat <30% of daily calorific intake. After randomisation all participants received lifestyle advice at three monthly intervals with emphasis on healthy cooking methods and adapting traditional recipes facilitated by the use of posters, photographs and a booklet on portion size.<br>**Intervention:**<br>dietary treatment with Orlistat 120 mg 3x daily before each meal (not included in this review)<br>**Theoretical framework**: NR<br>**Control:** dietary treatment alone	**Reported:**<br>% body fat<br>BP<br>BMI<br>Glucose<br>Insulin<br>Lipids<br>Protein<br>Waist circ<br>Weight<br>**Subgroup analysis by SES:** No	**Funding of intervention:** Roche Pharmaceuticals **Funding of evaluation:** NR<br>**Who delivered:** dietician<br>**Other resources:** NR<br>**Economic evaluation:** NR<br>**Process evaluation:** NR

Table S2. *Cont.*

Study ID	Methods	Participants	Intervention	Outcomes	Implementation
Ghosh 2006 [12]	**Design:** single-arm before and after study<br>**Aim:** To study the effect of brisk walking on physiological and obesity measures<br>**Duration:** 20 weeks<br>**Power:** NR<br>**ITT:** No	**Age (years):** 60.4 (6.2), 55–64<br>**Sex (M/F):** 100% M<br>**South Asian:** 100% occupants of Calcutta or its suburbs<br>**Baseline BMI:** 26.3 (1.2)<br>**Health status:** Overweight otherwise healthy<br>**Number:** 45<br>**Setting:** Visva Bharati University<br>**Country:** India	**Intervention:** 30-min daily walking at a stretch with moderate sweating (2 km) 20-week observation period was divided into four groups retrospectively (all participants completed 20-weeks):<br>group I (up to 5 weeks)<br>group II (6–10 weeks)<br>group III (11–15 weeks)<br>group IV (16–20 weeks)<br>**Theoretical framework**: NR<br>**Control:** None	**Reported:**<br>% Body fat<br>BP<br>BMI<br>Frequency of walking<br>Glucose<br>WHR<br>**Subgroup analysis by SES:** No	**Funding of intervention:** NR<br>**Funding of evaluation:** NR<br>**Who delivered:** NR<br>**Other resources:** NR<br>**Economic evaluation:** NR<br>**Process evaluation:** NR
Gulati 2014 [13]	**Design:** RCT treated as single-arm before-after study for this review<br>**Aim:** To evaluate effects of pistachio nuts on cardio-metabolic risk factors<br>**Duration:** 6-months<br>**Power:** yes, assuming mean (standard deviation) reduction in waist circ in the diet and exercise group as 2.0 (0.5) and expecting an additional reduction of 25% (2.5,sd 0.6) due to inclusion of	**Age (years):** 42.5 (8.2)<br>**Sex (M/F):** 37/31<br>**South Asian:** 100% Asian Indians<br>**Baseline BMI**: 30.9 (7.5)<br>**Health status:** MetS<br>**Number:** 68<br>**Setting:** Hospital<br>**Country:** Dehli, India	**3-week run in:** all participants received standard diet—60% carbohydrates, 15% protein, and 25% fat;<br>**Intervention:** pistachio diet: 51% carbohydrates, 20% protein, and 29% fat. Pistachios were substituted for visible fat (cooking oil and butter), a portion of carbohydrates, and dairy. Participants were advised to take pistachios as 20% of total energy.<br>**Theoretical framework**:	**Reported:**<br>Adinopectin<br>Adipose tissue<br>Free fatty acid<br>Glucose<br>Haemoglobin<br>Insulin<br>Leptin<br>Lipids<br>Protein<br>Thiobarbituric acid reactive substances<br>Tumor necrosis factor<br>Waist circ<br>Weight	**Funding of intervention:** Paramount Farms Inc., California, USA.<br>**Funding of evaluation:** NR<br>**Who delivered:** NR<br>**Other resources:** NR<br>**Economic evaluation:** NR<br>**Process evaluation:** compliance checks

Table S2. *Cont.*

Study ID	Methods	Participants	Intervention	Outcomes	Implementation
	pistachios to detect the above difference with 95% CI and 90% power, 26 participants were required in each arm. **ITT:** yes (after run-in) but RCT treated as single-arm before-after study for this review		NR **Control:** standard diet according to dietary guidelines for Asian Indians—60% carbohydrates, 15% protein, and 25% fat; for weight maintenance **All participants:** instructed to maintain the same level of PA. Advised to eat a diet rich in vegetables and fruits; select whole-grain, high-fiber foods; limit red meat and meat products, and use white meats instead; select fat-free or low-fat dairy products; limit foods containing partially hydrogenated vegetable oils; curtail consumption of soft drinks and foods with added sugar; choose and prepare foods with little or no salt; and limit alcohol intake. Diet counselling for individuals and groups were given every 30 days for first 4 months then once every 45 days for next 2 months.	**Subgroup analysis by SES:** No	

Table S2. *Cont.*

Study ID	Methods	Participants	Intervention	Outcomes	Implementation
Johnston 2013 [14]	**Design:** Cluster RCT<br>**Aim:** To slow the rate of weight gain in children<br>**Duration:** 2 years<br>**Power:** NR<br>**ITT:** Yes but not for South Asian overweight subgroup	**Age (years):** 7.8 (0.4)<br>**Sex (M/F):** 38% F<br>I (overweight/obese): 38% F; C (overweight/obese): 46%F; I (normal weight): 47% F; C (normal weight): 54% F;<br>**Baseline BMI:** overweight/obese (BMI ≥ 85th percentile): I: 21.6 (3.9); C: 21.0 (2.6); normal-weight (BMI ≥ 5th percentile and <85th percentile): I: 15.8 (1.1); C: 16.0 (1.1)<br>**South Asian:** 25.3% Asian<br>**Health status:** 321 overweight/obese (BMI ⩾ 85th percentile), 477 normal-weight (BMI ⩾ 5th percentile and <85th percentile), and 37 underweight (BMI<5th percentile), underweight excluded from analyses<br>**Number:** 835<br>**Setting:** 7 elementary schools, large suburban independent school district<br>**Country:** southwest of Houston, Texas, USA	**Intervention:** integrated health and physical education into existing school core curriculum using MI to address resistance to change. Trained health professional assisted teachers in creating and implementing lesson plans incorporating healthy messages. Health professionals worked with school administration, cafeteria staff and elective teachers to create a healthier school environment.<br>**Theoretical framework**: Motivational interviewing<br>**Control:** self-help condition where schools received the same curriculum materials and were encouraged to incorporate healthy messages into their existing curricula. No additional training or support was provided.	**Reported:**<br>Academic grades<br>BMI (kg/m2)<br>Odds Ratio of becoming overweight/obese at 2 years across treatment conditions and ethnic groups<br>Weight<br>zBMI<br>**Subgroup analysis by SES:** No	**Funding of intervention:** NR<br>**Funding of evaluation:** NR<br>**Who delivered:** Teachers assisted by trained health professionals, 20 h of didactic training, 40 h of in vivo training, and 40 h of supervised practice. Weekly supervision with 2 clinical psychologists and a registered dietician for 60 min<br>**Other resources:** All school staff involved<br>**Economic evaluation:** NR<br>**Process evaluation:** treatment fidelity

Table S2. *Cont.*

Study ID	Methods	Participants	Intervention	Outcomes	Implementation
Kameswararao 2009 [15]	**Design:** 4 group, before and after study treated as single-arm (obesity prevention) before-after study for this review<br>**Aim:** to reduce lifestyle risk factors for obesity<br>**Duration**: 6 months<br>**Power:**NR<br>**ITT:** No	**Age (years):** NR, school children<br>**Sex (M/F):** boys urban:31 rural: 5 = 36 total<br>**% South Asian:** 100%<br>**Baseline BMI:** NR, (n = 59/610 (9.7%) BMI ⩾95th percentile)<br>**Health status:** obese (BMI ⩾ 95th percentile)<br>**Number:** 610<br>**Setting**: school health clinics in health centres, Rampur urban and Vutoor rural schools (two clusters), Karimanagar district<br>**Country:** India	**Intervention:**<br>Children divided into 4 groups depending on need. Each team was assigned one intervention:<br>1. Obesity prevention and reduction<br>2. Prevention of excessive of sweets, chocolates and carbohydrate consumption<br>3. Reducing the duration of daily TV watching<br>4. Increasing PA<br>Each team conducted both theoretical and practical teaching sessions using audiovisual aids (2 h per week for 6 months).<br>**Obesity prevention and reduction group:** All the obese children were educated regarding their risks for diabetes. PA and diet modifications. Teachers, parent and community leaders were made aware of natural histories of childhood obesity, diabetes and their prevention and correction. Mothers and teachers were	**Reported:**<br>BMI<br>Diabetes<br>Obesity<br>PA<br>Sweet consumption<br>TV viewing<br>**Subgroup analysis by SES:** No	**Funding of intervention:** NR<br>**Funding of evaluation:** NR<br>**Who delivered:** 4 School Health Action teams. Each team led by two doctors comprised the children at risk, their parents and their class teacher and a local leader.<br>**Other resources: NR**<br>**Economic evaluation:** NR<br>**Process evaluation:** After study completion, school health clinics functioning at urban and rural health centres, headed by pediatrician was made responsible for the sustenance of the benefits and effects on an ongoing basis. Obesity clinics were commenced where there were more obese children. Diet plans, exercise plans, counselling and long term follow up sessions were carried out. Pediatric weight management programmes have been launched in these two areas.

Table S2. *Cont.*

Study ID	Methods	Participants	Intervention	Outcomes	Implementation
			advised to encourage the children to play outdoor games and prevent them watching TV for longer periods. Mothers were advised to be very cautious while feeding their children. They were advised not to over feed their children with carbohydrate diet. Parents with a history of diabetes were advised to be cautious while feeding their children. Urban rich families advised to avoid feeding chocolates to their children. **Theoretical framework**: NR **Control**: None		

Table S2. *Cont.*

Study ID	Methods	Participants	Intervention	Outcomes	Implementation
Khaskheli 2013 [16]	**Design:** single-arm before and after study<br>**Aim:** To assess the effect of weight reduction in obese infertile women on conception rate<br>**Duration:** 1 year, BMI measured at 6-months<br>**Power:** NR<br>**ITT:** No	**Age (years):** 31.06 (2.48)<br>**Sex (M/F):** 100% F<br>**South Asian:** 100%<br>**Baseline BMI:** 36.21 (1.35)<br>**Health status:** women who had taken treatment of infertility for 2–5 years and failed to conceive, BMI >30<br>**Number:** 98<br>**Setting:** private clinics at Mirpurkhas, Thana Bola Khan and Hyderabad<br>**Country:** Sindh, Pakistan	**Intervention:** Stopped conventional medical treatment for infertility during the study period. Fortnightly programme of lifestyle change (exercise and dietary habits) for 6 months, then were followed monthly for next 6 months. Those women who failed to conceive were prescribed ovulation induction (clomifene citrate) for the next 6 months and were observed for conception.<br>**Theoretical framework**: NR<br>**Control:** None	**Reported:**<br>BMI<br>Conception with ovulation induction<br>Pregnancy outcomes<br>Spontaneous conception<br>**Subgroup analysis by SES:** No	**Funding of intervention:** NR<br>**Funding of evaluation:** NR<br>**Who delivered:** NR<br>**Other resources:** NR<br>**Economic evaluation:** NR<br>**Process evaluation:** NR
Kousar 2008 [17]	**Design:** single-arm before and after study<br>**Aim:** To test the effectiveness of a diet and lifestyle intervention to treat components of MetS (including obesity)<br>**Duration:** 24-weeks<br>**Power:** calculated to detect a difference due to the intervention of a 9% fall in systolic	**Age (years):** 37.6 (4.3)<br>**Sex (M/F):** 100% F<br>**South Asian:** 100% married with children, Pakistani born female immigrants (in Australia for 5–17 years)<br>**Baseline BMI:**29.2 (SE 0.46)<br>**Health status:** at least 1 component of MetS: elevated BP, elevated	**12-week run-in period:** no contact<br>**Intervention:** 12-week culturally appropriate diet and lifestyle intervention. Female facilitator led small groups of migrant women through a specially designed educational and behavioural 'Step to good health program'.1Goal to decrease in energy intake	**Reported:**<br>BP<br>BMI<br>Glucose<br>Insulin<br>Lipids<br>PA<br>**Subgroup analysis by SES:** No	**Funding of intervention:** NR<br>**Funding of evaluation:** NR<br>**Who delivered:** female researcher, bilingual Pakistani female facilitator who was a trained nutritionist with expertise in obesity management<br>**Other resources:** NR<br>**Economic evaluation:** NR<br>**Process evaluation:** NR

Table S2. *Cont.*

Study ID	Methods	Participants	Intervention	Outcomes	Implementation
	BP, on the basis of a previous pilot study<br>**ITT:** No	blood glucose levels, obesity, or increased waist circ<br>**Number:** 53<br>**Setting:** University<br>**Country:** Melbourne, Australia	and increase in PA.<br>12 weekly modules consisted of individual dietary counselling and researcher-participant interaction. Participants were counselled for 4 h per week. Three hours one-to-one interaction, one hour telephone contact. Participants were requested to take 10,000 steps in 1 period (approximately 30–40 min of brisk walking) of PA, 6 days a week.<br>**Theoretical framework**: ‘Cultural competence’, also peer education model<br>**Control:** participants acted as own control during 12-week run-in period		

Table S2. *Cont.*

Study ID	Methods	Participants	Intervention	Outcomes	Implementation
Madsen 2009 [18]	**Design:** single arm before and after study<br>**Aim:** to evaluate the SCORES programme to reduce obesity in minority school children<br>**Duration:** 8-months<br>**Power:** NR, but authors report the greatest impact of lack of follow-up in this study is likely a decrease in power to see changes in BMI z-score<br>**ITT:** No	**Age (years):** 9.8 (0.9)<br>**Sex (M/F):** 86 of 178 were female (48% F)<br>**% South Asian:** 26% Asian (46/178)<br>**Baseline BMI:** 20.9 (4.9)<br>**Baseline BMI z-score:** 1.01 (1.05)<br>**Health status:**<br>18% of all children were overweight and 34% were obese. Asian boys had the highest prevalence of obesity (44%)<br>**Number:** 233<br>**Setting:** 9 primary schools<br>**Country:** San Francisco, USA	**Intervention:**<br>For eight weeks in the fall and ten weeks in the spring, children in the programme play soccer 3 days a week (two practice days with up to 2 h of moderate-to-vigorous PA and 1 inter-school game day with a warm-up period followed by a 1-hour game) and performing community service or creative writing the remaining two days a week.<br>In the spring, each team picks a community service project, which they execute under the guidance of their writing coach. Such projects have included community food, clothing, and toy drives, and neighbourhood beautification activities. The programmes curriculum, focused on literacy, leadership and teamwork skills, is identical for both genders,	**Reported:**<br>BMI<br>PA<br>**Subgroup analysis by SES:** No	**Funding of intervention:** part of its funding from the Cities of San Francisco and Oakland, with the balance coming from corporate and individual donors<br>**Funding of evaluation:** NR<br>**Who delivered:** Separate coaches for the soccer and literacy curricula, most of whom are teachers during the day at the same schools where they coach for SCORES. Staff members were trained in data collection and collected all anthropometric and fitness data and provided investigators at the University of California, San Francisco with a de-identified data set.<br>**Other resources:**<br>This is a pilot study of a pre-existing community-based programme: America SCORES started in 1994 and is now nationwide. San Francisco Bay Area SCORES is conducted in 24 public primary schools selected for having students with low academic performance and a high percentage of low-income families.<br>**Economic evaluation: NR**<br>**Process evaluation:** yes, attendance, also paper reports: coaches found it difficult to incorporate measures into busy and exciting game days (when most measures were scheduled in order to centralize the evaluation process). They also reported that a lack of coordination with schools (who scheduled field trips and special events on data collection

Table S2. *Cont.*

Study ID	Methods	Participants	Intervention	Outcomes	Implementation
			but all activities are conducted separately for girls and boys to best match sports skills.<br>**Theoretical framework:** NR<br>**Control:** None		days) hindered the evaluation. Finally, discipline issues leading to short-term suspension from SCORES for some children contributed to incomplete follow-up. Although participants were volunteers, SCORES provided free after-school care.
Mathews 2007 [19]	**Design:** in-service evaluation of a service development (one group, before and after study)<br>**Aim:** To manage cardiovascular risk factors<br>**Duration:** 6–12 months<br>**Power:** NR<br>**ITT:** No	**Age (years):** 44.45 (13.56)<br>**Sex (M/F):** 103 M/201 F<br>**South Asian:** Bangladeshi (*n* = 65, 21%), Indian (*n* = 71, 23%), Pakistani (*n* = 146,48%), other South Asian (*n* = 22, 8%)<br>**Baseline BMI:** 28.6<br>**Health status:** CHD risk factors, 48% family history of diabetes<br>**Number:** 304<br>**Setting:** clinics run from project work base, worksites, community venues including religious buildings.<br>**Country:** Edinburgh, Scotland, UK	**Intervention:** Health visitor led screening clinic with optional community activities.<br>30-minute initial screening followed by a further 30-minute consultation, (1–2 weeks later), to discuss blood tests, individual heart health profiles (CHD risk) and goal setting (baseline visit). Information offered on the Khush Dil community activity programme. Dietetic clinics provided one-to-one nutritional support. Practical activities including cookery workshops, exercise classes and CHD/diabetes awareness sessions to encourage lifestyle change and reduce CHD risk<br>**Theoretical framework**: Transtheoretical Model<br>**Control**: None	**Reported:**<br>BP<br>BMI<br>CHD risk score<br>Diet<br>Lipids<br>Motivational stage<br>PA<br>Waist circ<br>Weight<br>**Subgroup analysis by SES:** No	**Funding of intervention:** NHS Lothian, LPCT Primary Care Development Fund, and Local Health Plan. Research staff supported through Professor Bhopal's funds.<br>**Funding of evaluation:** NR<br>**Who delivered:** health visitor, dieticians, South Asian community workers, interpretation and translation supplied via the local council service or South Asian Health Workers<br>**Other resources:** Local GP helped adapt protocol for management of risk factors. Training was provided to project staff by a cardiac rehabilitation nurse and the project leader. Minor medical queries were directed to a doctor who offered the project support on a consultation basis.<br>**Economic evaluation:** set up on a 1-year health service innovations grant of about £50 000<br>**Process evaluation:** separate qualitative evaluation

Table S2. *Cont.*

Study ID	Methods	Participants	Intervention	Outcomes	Implementation
Misra 2008 [20]	**Design:** single-arm before and after study<br>**Aim:** To evaluate the effect of supervised progressive resistance-exercise training (PRT)<br>**Duration:** 12 weeks<br>**Power:** NR<br>**ITT:** No	**Age (years):** 40.8 (8.1)<br>**Sex (M/F):** 22 M/8 F<br>**% South Asian:** 100%<br>**Baseline BMI:** 24.1 (3.9) range 17.5 to 30.0<br>**Health status:** type 2 diabetes (10 hypertensive, 11 diabetic retinopathy)<br>**Number:** 30<br>Hospital, **Country:** New Delhi, India	**Intervention:** onsite supervised PRT 3 days per week in the physiotherapy clinic supervised by the same physiotherapist. 6 muscle groups (2 sets, 10 repetitions each): biceps flexion, shoulder flexion, finger grip, hip flexion, knee extension, and heel rise. If the patient was able to perform such exercise at the end of the week, 0.5 kg weight was added in the next week. Patients did not change the dose of oral hypoglycemic medications, dietary pattern, or intensity of baseline activities including aerobic exercises. Most patients were already following an aerobic exercise regimen as prescribed by their physicians, which was further reinforced.<br>**Theoretical framework**: NR<br>**Control**: None	**Reported:**<br>% body fat<br>BMI<br>Glucose<br>Glycated haemoglobin<br>Hip circ<br>Insulin tolerance test<br>Lean body mass<br>Lipids<br>Mid- arm circ<br>Mid- thigh circ<br>Muscle area<br>Regional fat<br>Skinfolds<br>Waist circ<br>WHR<br>**Subgroup analysis by SES:** No	**Funding of intervention:** partially supported by a grant from Sanofi-Aventis (India)<br>**Funding of evaluation:** NR<br>**Who delivered:** physiotherapist (an author), research staff<br>**Other resources:** NR<br>**Economic evaluation:** NR<br>**Process evaluation:** Compliance was observed at 85% through a self-maintained diary

Table S2. *Cont.*

Study ID	Methods	Participants	Intervention	Outcomes	Implementation
Nidhi 2012 [21]	**Design:** RCT<br>**Aim:** To assess the effect of yoga therapy on glucose metabolism an lipids<br>**Duration:** 12 weeks<br>A sample size of 80 participants was found to be adequate for an effect size of 0.63, an α level of 0.05, and a power of 0.80 (for fasting insulin outcome).<br>**ITT:** No	**Age (years):** 16.22 (15–18)<br>**Sex (M/F):** 100% F<br>**% South Asian:** 100%<br>**Baseline BMI:**I:20.30 (1.92); C: 21.22 (2.99)<br>**Health status:** polycystic ovary syndrome (PCOS), 85% normal-weight (BMI 18.5–23)<br>**Number:** 90<br>**Setting:** residential school<br>**Country:** Anantpur, Andhra Pradesh, India	**Intervention:** Both groups went through their respective set of practices 1 h per day each day for 12 weeks under the supervision of trained instructors, for a total of 90 sessions.<br>**Intervention:** Yoga, concepts from traditional yoga texts (Patanjali yoga sutras, Upanishads, and Yoga Vasishtha) that place emphasis on a holistic approach to health management at the physical, mental, emotional, and intellectual levels. The practices consisted of asanas (yoga postures), pranayama, relaxation techniques, and meditation, along with lectures on yogic lifestyle and yogic counselling for stress management. All adolescents received at least 1 session, lasting about 1 h, of individualized counselling aiming at cognitive	**Reported:**<br>BMI<br>Glucose<br>Hip circ<br>Insulin<br>Lipids<br>Waist circ<br>WHR<br>**Subgroup analysis by SES:** No	**Funding of intervention:** Central Council for Research in Yoga and Naturopathy, Ministry of Health, Government of India, Central Trial Registry of India No: REFCTRI-2008 000291<br>**Funding of evaluation:** NR<br>**Who delivered:** Two parallel training modules, one for each group, were developed by a team of experts that included a psychiatrist, a gynaecologist, and a yoga therapy physician. A medical officer, ultrasound specialist, laboratory staff (blinded to the groups), statistician performed the randomization and final analysis (blinded to data source).<br>**Other resources:** NR<br>**Economic evaluation:** NR<br>**Process evaluation:** NR

Table S2. *Cont.*

Study ID	Methods	Participants	Intervention	Outcomes	Implementation
			restructuring based on yoga philosophy<br>**Theoretical framework:** NR<br>**Control:** conventional physical exercises and safe nonyogic breathing followed by supine rest (without instructions). Also received 1 counselling session. Care was taken by the counsellors not to introduce any of the yogic concepts during these sessions.		
Prabhakaran 2009 [22]	**Design:** 2 group B & A (subset of repeat cross-sectional study)<br>**Aim:** To assess CVD prevention programme<br>**Duration:**3.7 to 4 years<br>**Power:** NR<br>**ITT:** No	**Age (years):** I: 40.8 (10.8); C:38.6 (11.7)<br>**Sex (M/F):** I: 58.7% M; C: 58.1% M<br>**South Asian:** 100% Indian<br>**Baseline BMI:** NR<br>**Health status:** general population (employees and their family members aged 10–69)<br>**Number:** 2331<br>**Setting:** 6 industrial sites<br>**Country:** India	**Intervention:**<br>Increase consumption of locally available fruits and vegetables and to move toward a healthy diet (higher fibre and decreased salt and oils). Incorporation of PA in daily living, avoidance of tobacco and maintenance of a healthy body weight. Improving awareness of need to treat high blood pressure and diabetes. Posters and banners,	**Reported:**<br>Blood pressure<br>Glucose<br>Lipids<br>Weight<br>Waist Circ<br>**Subgroup analysis by SES:** No	**Funding of intervention:**<br>World Health Organization,<br>New Delhi, and the Ministry of Health and Family<br>Welfare, Government of India<br>**Funding of evaluation:** NR<br>**Who delivered:** trained, locally stationed, project health care personnel<br>**Other resources:** Risk stratification and treatment guidelines for diabetes and hypertension were prepared for on-site physicians<br>**Economic evaluation:** NR<br>**Process evaluation:** NR

Table S2. *Cont.*

Study ID	Methods	Participants	Intervention	Outcomes	Implementation
			handouts, booklets, and real-time videos were translated into 7 Indian languages.<br>Direct one-on-one interactions including family, and group sessions.<br>Canteen menu modified, onsite smoking banned.<br>Adults with risk factor referred to a health care facility for further risk management and provided with individual and group counselling on diet, smoking, PA.<br>**Theoretical framework**: socioecological model, social cognitive theory of behaviour change and social learning theory<br>**Control:** individuals with established CVD risk factors were referred to the industry-managed clinic; 3 human immunodeficiency virus/acquired immunodeficiency syndrome awareness programs and a health talk on smoking. Smoking banned inside work.		

Table S2. *Cont.*

Study ID	Methods	Participants	Intervention	Outcomes	Implementation
Ramachandran 2006 [23]	**Design:** RCT<br>**Aim:** To determine whether interventions could modify incidence of type 2 diabetes<br>**Duration:** 36 months<br>**Power:** assumption of a 30% cumulative incidence of type 2 diabetes over 3 years in control and 50% reduction with intervention, at 5% significance with 80% power, allowing 10% dropout, 134 participants per group were needed<br>**ITT:** No	**Age (years):** I: 46.1 (5.7); C:45.2 (5.7)<br>**Sex (M/F):** I: 78% M; C: 76% M<br>**South Asian:** 100% Asian Indian<br>**Baseline BMI:** I: 25.7 (3.3); C: 26.3 (3.7)<br>**Health status:** impaired glucose tolerance<br>**Number:** 269<br>**Setting:** community, middle-class population and families working in service organisations<br>**Country:** India	**Intervention:** healthy diet (reduction calories, refined carbohydrates and fats, avoidance of sugar, inclusion of fibre-rich foods) and regular PA. Participants involved in physical labour or who had to walk or cycle for >30 min/day or were performing exercises regularly were asked to continue their routine activities. Participants engaged in sedentary or light PA were advised and regularly motivated to walk briskly for at least 30 min each day.<br>**Theoretical framework**: NR<br>**Control:** standard healthcare advice	**Reported:**<br>Adherence to diet & PA,<br>Adverse events<br>BMI<br>Incidence diabetes<br>Waist Circ<br>Weight<br>**Subgroup analysis by SES:** No	**Funding of intervention:**<br>M/S US Vitamins<br>**Funding of evaluation:** NR<br>**Who delivered:** physician, 3 laboratory technicians, a dietician, a social worker and a helper<br>**Other resources:** internal safety committee including administrator, legal advisor, consultant physician<br>**Economic evaluation:** NR<br>**Process evaluation:** NR

Table S2. *Cont.*

Study ID	Methods	Participants	Intervention	Outcomes	Implementation
Ramachandran 2013 [24]	**Design:** RCT<br>**Aim:** To assess whether mobile phone messaging lifestyle advice could reduce incident type 2 diabetes<br>**Duration:**20.2 months<br>**Power:** assumption of a 30% cumulative incidence of type 2 diabetes over 2 years, at 5% significance with 80% power, 214 participants per group were needed for a 40% reduction in progression to type 2 diabetes to be detected.<br>**ITT:** Yes	**Age (years):** I: 45.9; C: 46.1<br>**Sex (M/F): 100% M**<br>**South Asian:** 100% Asian Indian<br>**Baseline BMI:** I: 25.8 (3.3); C: 25.8 (3.0)<br>**Health status:** Impaired glucose tolerance<br>**Number:** 537<br>**Setting:** 10 public-sector and private-sector industrial units<br>**Country:** southeast India (Chennai, Tamil Nadu and Visakhapatnam, Andhra Pradesh).	**Intervention:** In addition to standard lifestyle modification advice, participants received mobile phone messages at frequent intervals containing information about healthy lifestyle, benefits of PA and diet, cues to start PA and healthy dietary practices, and strategies to avoid relapse and remain motivated to maintain PA and healthy dietary habits. Participants would not be likely to receive the same message in a 6-month period (on the basis of them receiving two to four messages per week). The timing (0500–0800 h or 1700–2000 h) and frequency of mobile phone messaging were tailored to the participants preferences<br>**Theoretical framework**: Transtheoretical model of behavioural change<br>**Control:** standard lifestyle modification advice at baseline only	**Reported:**<br>Blood pressure<br>BMI<br>Dietary intake<br>Lipids<br>PA<br>Type II diabetes<br>Waist circ<br>**Subgroup analysis by SES:** No	**Funding of intervention:**<br>UK–India Education and Research Initiative (UKIERI), World Diabetes Foundation<br>**Funding of evaluation:** The evaluation board of the UKIERI assessed the outline protocol in a competitive funding process, but neither UKIERI nor the World Diabetes Foundation had a role in study design, data collection, data analysis, data interpretation, or writing of the report.<br>**Who delivered:** The messages were delivered by a commercial service Provider (Unicel technologies, India).<br>**Other resources:** mobile phone messaging delivery manager website was created in partnership with Intel (Bangalore, India).<br>**Economic evaluation:** NR<br>**Process evaluation:** acceptability

Table S2. *Cont.*

Study ID	Methods	Participants	Intervention	Outcomes	Implementation
Rush 2007 [25]	**Design:** single-arm before and after study<br>**Aim:** To assess the composition, blood lipid profile and insulin resistance in migrant Asian effect of lifestyle intervention<br>**Duration:** 5 months<br>**Power:** NR<br>**ITT:** No	**Age (years):** >50;M 62 (8); F59 (8)<br>**Sex (M/F):** 21 M/20 F<br>**% South Asian:** 100% Asian Indians resident in urban Auckland, volunteers<br>**Baseline BMI:** NR<br>**Health status:** healthy, >50 years<br>**Number:** 41 completers<br>**Setting:** Department of surgery body composition laboratory, and community<br>**Country:** Auckland, New Zealand	**Intervention:** group PA and diet education. Participants received 2 baseline visits one month apart. An initial group education session to encourage increased PA and improved diet was delivered to two community groups at their regular meeting. Two booklets were provided (one with Hindi translation). Those who had undergone the two baseline measurements were provided with individually- marked waist threads (to assess changes in girth), pedometers and diaries to record number of steps taken daily. Following this initial session, monthly group sessions with handouts were held which included a cooking demonstration with encouragement to use canola oil in place of other oils, to remove fat from meat, and to increase fish consumption; a pedometer club; and “weigh-ins”.<br>**Theoretical framework:** NR<br>**Control**: None	**Reported:**<br>Abdominal fat<br>BP<br>Body fat<br>Glucose<br>Insulin<br>Lipids<br>Waist circ<br>Weight<br>**Subgroup analysis by SES:** No	**Funding of intervention:** Auckland University of Technology Contestable Research Fund.<br>**Funding of evaluation:** NR<br>**Who delivered:** NR<br>**Other resources:** A booklet was written by the first author was written especially for the study: *Healthy Living*, *Putting the squeeze on lifestyle disease for NZ Indian people*<br>**Economic evaluation:** NR<br>**Process evaluation:** NR

Table S2. *Cont.*

Study ID	Methods	Participants	Intervention	Outcomes	Implementation
Shailaja 2011 [26]	**Design:** 2 group before and after study<br>**Aim:** To evaluate the effectiveness of patient education on weight reduction<br>**Duration:** 3-months<br>**Power:** NR<br>**ITT:** No	**Age (years):** 18–60<br>**Sex (M/F):** 81 M/19 F (urban); 46 M/17 F (rural)<br>**South Asian:** 100%<br>**Baseline BMI:**<br>Urban: males:28.09; females: 29.26;<br>Rural: males: 27.11; females: 28.18<br>**Health status:** BMI 25–40<br>**Number:** 200 (100 urban, 100 rural)<br>**Setting:** urban community pharmacy in Abhiramapuram, Chennai; Free obesity-awareness camps in T.Nagar, Chennai (urban) and Odhiyathur, Villupuram district (rural)<br>**Country:** Tamil Nadu, India	**Intervention:** counselling on diet, PA and behavioural modifications. Booklets "Lifestyle Modifications" and "South Indian Food Items and their Calories" were provided which contain guidelines for selection of food items (low calorie, low fat alternatives, and food exchange list), guidelines for PA and behaviour change, both in English and Tamil. Participants were advised not to take more than three meals and 1–2 snacks per day. They were asked to take the normal food as prepared for the rest of the family members and avoid high caloric food items. Participants were asked to walk briskly at least 30 min a day. They were recommended to consult a dietician and fitness instructor for effective weight reduction. Reviewed at least once in a month.<br>**Theoretical framework**: NR<br>**Control**: None	**Reported:**<br>% overweight/obese<br>Awareness of overweight/obesity<br>BMI<br>Lifestyle (smoking, alcohol use)<br>Waist circ<br>Weight<br>**Subgroup analysis by SES:** No	**Funding of intervention:** NR<br>**Funding of evaluation:** NR<br>**Who delivered:** community pharmacist<br>**Other resources:** booklets<br>**Economic evaluation:** NR<br>**Process evaluation:** NR

Table S2. *Cont.*

Study ID	Methods	Participants	Intervention	Outcomes	Implementation
Sharma 2009 [27]	**Design:** Retrospective audit (4 groups)<br>**Aim:** To determine effectiveness of Ayurvedic diets<br>**Duration:** 3 months<br>**Power:** NR<br>**ITT:** No	**Age (years):** 20–60<br>**Sex (M/F):** 24 M/176 F<br>**South Asian:** 100%<br>**Baseline BMI:** 31.50 (5.15)<br>**Health status:** BMI > 25kg/m^2<br>**Number:** 200<br>**Setting:** Ayurvedic clinics<br>**Country:** South Delhi, India	**Intervention:** Diet based on the Ayurvedic constitution, based on the predominant *doshas. For people of*<br>1. *vatta* constitution, diets based mainly on wheat, potato, rice, black gram, besan (gram flour), and fish<br>2. *pitta* constitution, diets based on barley, besan (gram flour), dairy *(paneer),* animal foods like chicken or egg, rice, amla, soya, and green gram<br>3. *kapha* constitution, diets based mainly on wheat bran, barley, soy nuggets, besan (gram flour), green gram, and garlic<br>**Theoretical framework**: Ayurvedic medicine<br>**Control:** No control	**Reported:**<br>Abdominal circ<br>Arm circ<br>BMI<br>Chest circ<br>Hip circ<br>Thigh circ<br>Waist circ<br>Weight<br>**Subgroup analysis by SES:** No	**Funding of intervention:** An author is Managing Director of NutriHealth Systems<br>**Funding of evaluation:** NR<br>**Who delivered:** Ayurvedic consultant, nutritionist<br>**Other resources:** NR<br>**Economic evaluation:** NR<br>**Process evaluation:** NR

Table S2. *Cont.*

Study ID	Methods	Participants	Intervention	Outcomes	Implementation
Singhal 2010 [28]	**Design:** RCT<br>**Aim:** To assess the effectiveness of a school-based low-cost nutrition and lifestyle education intervention on behaviour modification and risk profile of Asian Indian adolescents<br>**Duration:** 6 months<br>**Power:** Estimated that 96 subjects in each group would be a sufficient sample size to achieve 5% target absolute reduction in BMI at a power of 80% and $p< 0.05$.<br>**ITT:** No	**Age (years): I:** 16.04 (0.41); C: 16.0 (0.5) (15–17)<br>**Sex (M/F)**: I: 60 M/39 F; C: 61 M/41 F<br>**South Asian:** 100%, schools 'matched for student strength and middle socioeconomic strata'<br>**Baseline BMI:** NR<br>**Health status:** healthy<br>**Number:** 201<br>2 co-educational schools<br>**Country:** Urban North India	The multi-component model included 7 components of nutrition and lifestyle education aimed at changing the knowledge, behaviour and risk profile of urban Asian Indian adolescents. (1) Dissemination of health related information through lectures and focused group discussions; (2) PA; (3) other healthy lifestyle activities; (4) individual counselling; (5) policy-level changes in school; (6) involvement of teachers and parents; (7) training of student volunteers to sustain programme<br>**Theoretical framework:** NR<br>**Control**: did not receive any intervention	**Reported:**<br>Arm, leg and trunk fat mass and muscle mass<br>BMI<br>Body image and self-esteem<br>Dietary intake<br>Glucose<br>Knowledge levels<br>Lipids<br>Mid-thigh circumference<br>Mid-upper arm circ<br>PA levels<br>Sagittal abdominal diameter<br>Tricep and bicep skinfold thicknesses<br>Waist Circ<br>Waist to thigh ratio<br>Waist-height ratio<br>Weight<br>WHR<br>**Subgroup analysis by SES:** No	**Funding of intervention:** World Diabetes Foundation, Denmark (WDF05-120).<br>**Funding of evaluation:** NR<br>**Who delivered:** nutritionist, physician<br>**Other resources:** TANITA CORPORATION (Tokyo, Japan) helped with the availability of TANITA MC-180MA, Dr Misra (co-author), Director, Lifeline Laboratory, conducted biochemical estimations<br>**Economic evaluation:** used peer educators and student volunteers to make programme sustainable and 'low-cost', nutritionist and a physician offered free of charge services at a health camp<br>**Process evaluation:** NR

Table S2. *Cont.*

Study ID	Methods	Participants	Intervention	Outcomes	Implementation
Telle-Hjellset 2013 [29] InnvaDiab-DE-PLAN study	**Design:** RCT<br>**Aim:** To improve risk factor profile for type 2 diabetes and MetS<br>**Duration:** 7 months<br>**Power:** Yes, calculated on the basis of a presumed 10% reduction in 2 h glucose, significance level of 5% and power of 80%.<br>**ITT:** No	**Age (years):** I: 41; C: 42<br>**Sex (M/F):** 100% F<br>**South Asian:** 100% Pakistani immigrant females (born in Pakistan or in Norway by 2 Pakistani parents)<br>**Baseline BMI:** I: 29.4; C: 29.8 (range 19.6 to 52.4)<br>**Health status:** adults with known type II diabetes excluded but identified in 12%–14% adults at baseline<br>**Number:** 198<br>**Setting:** local mother and child health clinic<br>**Country:** Oslo, Norway	**Intervention:** culturally adapted education programme. 9 subgroups of 10–12 women were offered six educational sessions, each lasting 2 h about blood glucose, PA and diet. Participants should obtain the necessary knowledge and understanding about how to influence their blood glucose level in everyday life. Focused on the effect of refined carbs (sugar, white bread and rice) on blood sugar, and health benefits of cutting down on these, and exchanging them with wholegrain foods.<br>Encouraged to walk for 1 h (approximately 5000 steps) 2x week. Child care offered, and baby-friendly walking paths. Walking shoes were provided.<br>**Theoretical framework**: education approach based on principals of empowerment. Stages of	**Reported:**<br>BP<br>BMI<br>C-peptide<br>Dietary intake<br>Glucose<br>Glycated Hb<br>Insulin<br>Intentions to change dietary behaviour<br>Intentions to reduce weight<br>Lipids<br>MetS<br>Waist circ<br>**Subgroup analysis by SES:** Yes in text, but data not shown	**Funding of intervention:** Norwegian Research Council (166977/166998), DE-PLAN Project (2004310), Norwegian Directorate of Health, Throne Holst Foundation (2875) and the Jahre Foundation.<br>**Funding of evaluation:** NR<br>**Who delivered:** Urdu-, Punjabi- and English-speaking research assistant was in charge of the recruitment. Urdu- and/or Punjabi-speaking Interviewers.<br>**Other resources:** Reebok provided shoes.<br>**Economic evaluation:** NR<br>**Process evaluation:** analysis by attendance

Table S2. *Cont.*

Study ID	Methods	Participants	Intervention	Outcomes	Implementation
			change construct from the Transtheoretical Model (TTM) was used to study the intentions to change dietary habits. **Control:** feedback on blood sugar levels, and lifestyle advice in one single short, group session		

Table S3. Study outcomes.

Study ID	Primary Outcomes	Summary
Adab 2014 [1]	**BMI z-score baseline:** I: −0.03 (1.37) *n* = 267; C: 0.08 (1.39) *n* = 304<br>**BMI z-score 2 years:** I: 0.13 (1.5) *n* = 234; C: 0.40 (1.5) *n* = 254<br>**2 yr BMI z-score between group difference (adjusted for baseline differences):** −0.15 (−0.26 to −0.03) *n* = 486<br>**2 yr BMI z-score between group difference (adjusted for baseline differences and age, sex, ethnicity):** −0.15 (−0.27 to −0.03) *n* = 486<br><br>**WC (cm) baseline:** I: 55.6 (7.7) *n* = 265; C: 55.3 (6.9) *n* = 304<br>**WC (cm) 2 years:** I: 59.4 (9.5) *n* = 228; C: 60.4 (9.1) *n* = 244<br>**2 yr WC (cm) between group difference (adjusted for baseline differences):** −0.88 (−1.87 to 0.10) *n* = 482<br>**2 yr WC (cm) between group difference (adjusted for baseline differences and age, sex, ethnicity):** −0.86 (−1.87 to 0.15) *n* = 482<br><br>**The risk of obesity was significantly lower in the intervention compared with the control group (OR 0.41; 95% CI 0.19 to 0.89).**	Adjusted between group differences at 2 year follow-up were significant in favour of intervention for BMI-z-score but no significant difference for WC. Unadjusted between group differences a 2 year follow-up were not significant for BMI z-score (reviewer calculated data). Other anthropometric changes between groups were not statistically significant.<br>The risk of obesity was significantly lower in the intervention compared with the control group (OR 0.41; 95% CI 0.19 to 0.89).

Table S3. *Cont.*

Study ID	Primary Outcomes	Summary
	Also reports bioimpedance, weight status, skinfolds<br>Multivariate analyses including only children of SA ethnicity: mean differences and ORs for the outcomes were of a similar magnitude to the main analyses (data not shown).<br>***Reviewer calculated data:***<br>***2 yr BMI z-score change: I: 0.16 (1.37) n = 234; C: 0.32 (1.39) n = 254***<br>***2 yr WC (cm) change:3.8 (7.7) n = 228; C: 5.1 (6.9)n = 244***<br>***Numbers of participants included in analyses confirmed with study authors***	
Admiraal 2013 [2]	**BMI (kg/m^2) baseline:** I: 28.1 (3.8) *vs*.. C: 27.2 (3.8)<br>**BMI (kg/m^2) 1 year:** I: 28.0 (4.0) *vs*.. C: 27.4 (3.8)<br>**1 year BMI (kg/m^2) change:** I: −0.1(1.2) *vs.* C:0.1(1.1)<br>**1 year BMI (kg/m^2) between group difference (95% CI): 0.2 (−0.04, 0.5)**<br><br>**WC (cm) baseline:** I: 94 (11) *vs.* C: 92 (10)<br>**WC (cm) 1 year:** I: 95 (10) *vs.* C: 94 (13)<br>**1 year WC (cm) change:** I:+1(5) *vs.* C: +2(10)<br>**1 year WC (cm) between group difference (95% CI):** 1 (−1, 2)<br><br>**WT (kg) baseline:** I: 76.3 (14.1) *vs.* C: 73.7 (12.7)<br>**WT (kg) 1 yr:** I: 76.1 (13.7) *vs.* C: 74.1 (12.9)<br>**1 year WT (kg) change:** I: −0.2(3.3) *vs.* C:0.4 (3.1)<br>**1 year WT (kg) between group difference (95% CI):** 0.6 (−0.1, 1.3)<br><br>**N:** I:177 *vs.* C:158	BMI, WC, WT not significantly improved at one year. Low response rate and high attrition.
Almas 2013 [3]	**BMI (kg/m^2) z-score:** Baseline: I: −1.35 (1.39) *vs.* C: −1.92 (1.82)<br>**BMI (kg/m^2) z-score:** 20-weeks: I: −1.10 (1.41) *vs.* C:−1.04 (1.23)<br>**BMI (kg/m^2) z-score:** change at 20-weeks:I −0.33 (1.20) *vs.* C: −0.88 (1.11)<br>**Difference in mean change from baseline to follow-up in BMI-z score:** 0.55 kg/m^2<br>**N:** I:105 *vs.* C:117<br>***Change data confirmed with study author***	Both intervention and control groups lost WT and control lost more WT than intervention. Limited generalisability due to convenience sampling and underpowered feasibility trial.

Table S3. *Cont.*

Study ID	Primary Outcomes	Summary
Andersen 2013 [4]	**BMI (kg/m^2) change at 5-months:** I: −0.5 (SE0.1); C: 0.3 (SE0.1)<br>**BMI (kg/m^2) adjusted mean difference (95% CI) at 5-months (adjusted for age and baseline differences):** −0.8 (−1.1 to −0.5)<br><br>**WC** (cm), change at 5 months: I: −1.9 (SE0.4) *vs.* C: +1.7 (SE0.4)<br>**WC (cm) adjusted mean difference (95% CI) at 5 months (adjusted for age and baseline differences):** I: −3.4 (−4.7 to −2.0)<br><br>**WT (kg) change at 5-months:** I: −1.7 (SE0.2); C: 0.1 (SE0.3)<br>**WT (kg) adjusted mean difference (95% CI) at 5-months (adjusted for age and baseline differences):** −1.9 (−2.7 to −1.0)<br><br>**N:** I: 76; C: 52 (same numbers assumed for BMI and WT as for WC)	BMI, WC, WT significantly lower after 5 months in the intervention group than in the control group. High quality study. Representative of Norwegian-speaking Pakistani immigrants.
Backes 2008 [5]	**BMI (kg/m^2):** Baseline: 30.2 (0.8)<br>**BMI (kg/m^2):** 3 months: 28.2 (0.8)<br><br>**WT (kg):** Baseline: 75.5 (1.7)<br>**WT (kg):** 3 months: 70.5 (1.6)<br>**N: 19**	BMI and WT were significantly lower at 3 months compared to baseline. Study sample unlikely to be representative, small study where 4/23 women who lost <5 pounds were excluded from analyses.
Balagopal 2008 [6]	**ADULTS:**<br>**BMI (kg/m^2):** Baseline: 20.6 (3.9)<br>**BMI (kg/m^2):** 7 months: 20.8 (3.9)<br>**WC (inches):** Baseline: 29.8 (4.1)<br>**WC (inches):** 7 months: 28.0 (4.3)<br>**N: 703**<br><br>**YOUTH (10–17):**<br>**BMI (kg/m^2):** Baseline: 16.0 (2.7)<br>**BMI (kg/m^2):** 7 months: 16.5 (2.4)<br>**WC (inches):** Baseline: 24.4 (2.9)<br>**WC (inches):** 7 months: 22.7 (2.5)<br>**N:112**<br>Also reports BMI and WC for IFG group and Diabetes subgroups.	WC but not BMI significantly reduced at follow-up. In youth, WC significantly reduced but BMI increased. Representative, community-based study.

Table S3. *Cont.*

Study ID	Primary Outcomes	Summary
Balagopal 2012 [7]	**BMI (kg/m^2):** Baseline: 20.73 (4.3)<br>**BMI (kg/m^2):** 6 months: 20.64 (4.2)<br>**Percentage change in BMI (kg/m^2)** at 6 months: −0.46<br><br>**WC (inches):** Baseline: 29.66 (4.8)<br>**WC (inches):** 6 months: 29.44 (4.8)<br>**Percentage change in WC (inches)** at 6 months: −1.25<br><br>**N:1638**<br>Also reports for Normal, IFG and Diabetes subgroups.<br><br>Also reports by high and low SES:<br>HIGH SES<br>**BMI (kg/m^2):** Baseline: 22.11 (4.6)<br>**BMI (kg/m^2):** 6 months: 21.97 (4.5)<br>**Percentage change in BMI (kg/m2)** at 6 months: −0.71, *n* = 874<br><br>**WC (inches):** Baseline: 30.94 (4.9)<br>**WC (inches):** 6 months: 30.76 (5.0)<br>**Percentage change in WC (inches)** at 6 months: −1.00, *n* = 874<br><br>LOW SES<br>**BMI (kg/m^2):** Baseline: 19.16 (3.5)<br>**BMI (kg/m^2):** 6 months: 19.13 (3.4)<br>**Percentage change in BMI (kg/m2)** at 6 months: −0.19, *n* = 764<br><br>**WC (inches):** Baseline: 28.19 (4.2)<br>**WC (inches):** 6 months: 27.92 (3.92)<br>**Percentage change in WC (inches)** at 6 months: −1.53, *n* = 764	Significant reduction in BMI and WC from baseline to 6-month follow-up. Relatively large, representative, community-based participatory study.<br>Subgroup analysis of BMI and WC by low and high SES. Only reports change from baseline to follow-up for high SES and for low SES (does not directly compare difference between high and low SES groups at follow-up). Both high and low SES groups showed significant reduction in BMI and WC at follow-up as measured by percentage change.

Table S3. *Cont.*

Study ID	Primary Outcomes	Summary
Bellary 2008 [8]	**BMI (kg/m^2): Difference between means (95% CI) Crude differences based on *t*-test comparison, no adjustment:**0.38 (0.20 to 0.55)<br>**BMI (kg/m^2): Difference least squares means (95% CI) Differences based on fixed effects model, adjusted for confounding (sex, age at diagnosis of diabetes, duration of diabetes, baseline differences):**0.40 (0.22 to 0.57)<br>**BMI (kg/m^2): Difference least squares means (95% CI) Differences based on mixed model, adjusted for confounding and clustering (sex, age at diagnosis of diabetes, duration of diabetes, baseline differences):**0.40 (0.20 to 0.60)<br><br>**WC (cm): Difference between means (95% CI) Crude differences based on *t*-test comparison, no adjustment:** −0.3 (−1.0 to 0.5)<br>**WC (cm): Difference least squares means (95% CI) Differences based on fixed effects model, adjusted for confounding (sex, age at diagnosis of diabetes, duration of diabetes, baseline differences):** −0.1 (−0.8 to 0.6)<br>**WC (cm): Difference least squares means (95% CI) Differences based on mixed model, adjusted for confounding and clustering (sex, age at diagnosis of diabetes, duration of diabetes, baseline differences):** −0.2 (−1.3 to 0.9)<br><br>***Additional data provided by authors:***<br>**Change in BMI (kg/m^2) at 24 months:** I: 0.31 (1.66); C: −0.07 (1.71) N: I: 861; C: 615<br>**Change in WC (cm) at 24 months:** I: 0.90 (7.16); C: 1.15 (7.28) N: I: 861; C: 618<br>**Change in WT (kg) at 24 months:** I: 0.50 (6.99); C: −0.38 (5.45) N: I: 867; C: 617	BMI and WT were significantly increased in the intervention group compared to the control group at 2 years. There was no significant difference in WC between the two groups at follow-up. Limited generalisability due to low response rate, otherwise good quality study.
Bhopal 2014 [9]	**BMI (kg/m^2) adjusted mean difference between groups at 3 years (adjusted for baseline differences and ethnicity and location):** I: −0.60 (−1.06 to −0.14)<br>**BMI (kg/m^2) baseline;** I: 30.59 (5.02); C: 30.49 (4.60);<br>**1 year;** I: 30.18 (5.04); C: 30.39 (4.56;<br>**2 years;** I: 30.31 (5.15); C: 30.57 (4.84);<br>**3 years;** I: 30.18 (5.50); C: 30.65 (4.83)<br><br>**WC (cm), adjusted mean difference between groups at 3 years (adjusted for baseline differences and ethnicity and location):** −1.89 (−3.27 to −0.52)<br>**WC (cm) baseline;** I: 102.69 (11.16); C: 103.26 (11.01);<br>**1 year;** I: 101.55 (11.34); C: 103.45 (11.66);<br>**2 years;** I: 102.04 (11.22);C:103.43 (11.19);<br>**3 years;** I: 100.51 (11.51); C:102.85 (11.14);<br><br>**WT (kg), adjusted mean difference between groups at 3 years (adjusted for baseline differences and ethnicity and location):** −1.64 (−2.83 to −0.44)	Significant adjusted difference between groups in favour of intervention for BMI, WC and WT at 3 years. Representativeness unclear, otherwise high quality RCT.

Table S3. *Cont.*

Study ID	Primary Outcomes	Summary
	**WT (kg) baseline;** I: 79.77 (16.23); C: 80.68 (14.98);<br>**1 year: I:** 78.82 (16.11); C: 80.36 (14.80);<br>**2 years; I:** 79.09 (15.94); C: 80.96 (15.10);<br>**3 years; I:** 78.76 (16.57); C: 80.99 (15.34);<br><br>Mean differences adjusted for ethnic group, city and baseline value.<br>**N:** I:84; C:83<br><br>***Reviewer calculated data:***<br>***3 yr BMI change: I: −0.41 (5.02) n = 84; C: 0.16 (4.60) n = 83***<br>***3 yr WC change: I: −2.18 (11.16) n = 84; C: −0.41 (11.01) n = 83***<br>***3 yr WT change: I: −1.01 (16.23) n = 84; C: 0.31 (14.98) n = 83***	
Chander 2013 [10]	**BMI (kg/m^2):** Baseline: 30.64 (3.63) n = 51<br>**BMI (kg/m^2):** 10 months: 29.04 (3.67) n = 51	BMI showed a significant decrease at 10-month follow-up. Low quality study. Study sample unlikely to be representative of general population, results pertain to highly compliant subgroup who also received usual drug treatment which may have influenced results.
Dixon 2008 [11]	**BMI (kg/m^2):** Baseline: 27.3 (3.1)<br>**BMI (kg/m^2):** 1 year: 26.8 (3.1)<br><br>**WC (cm):** baseline: 98.8 (9.0)<br>**WC (cm)**: 1 year: 96.7 (7.2)<br><br>**WT (kg):** baseline: 72.9 (9.9)<br>**WT (kg):** 1 year: 71.9 (9.8)<br><br>**N:** 16; Intervention arm not data extracted.	Only the control group (diet) is included in this review. BMI but not WT or WC was significantly reduced at 1 year compared to baseline. Small study sample unlikely to be representative of general population.

Table S3. *Cont.*

Study ID	Primary Outcomes	Summary
Ghosh 2006 [12]	**BMI (kg/m^2):** Baseline: 26.3 (1.2)<br>**BMI (kg/m^2):** 20-weeks: 24.8 (1.2)<br>**N:** 45	BMI significantly reduced at 20-week follow-up compared to baseline. Small study sample not representative of general population.
Gulati 2014 [13]	**WC (cm): Baseline:**; C: 106.0 (7.9);<br>**WC (cm): 24-weeks:** C: 102.5 (2.6);<br><br>**WT (kg): Baseline:** C: 80.3 (10.3);<br>**WT (kg): 24-weeks:** C: 78.9 (2.1);<br><br>**N:** 35; Intervention arm not data extracted.	Only the control group (diet) is included in this review. Study only reports difference between groups not significance from baseline to follow-up so significance of results for control group are unclear. However, control group lost 3.5cm WC and lost 1.4 kgs weight. Small study sample unlikely to be representative of general population.
Johnston 2013 [14]	**OverWT/obese subgroup:**<br>**BMI (kg/m^2): Change from baseline to 12-months:** I: 0.80 (1.30) *vs.* C: 0.78 (1.40)<br>**BMI (kg/m^2): Change from baseline to 24-months:** I: 1.67 (1.67) *vs.* C: 1.92 (1.91)<br><br>**zBMI: Change from baseline to 12-months: I:** −0.07 (0.24) *vs.* C: −0.05 (0.25)<br>**zBMI: Change from baseline to 24-months:** I: −0.08 (0.27) *vs.* C: −0.02 (0.27)<br><br>**WT: change from baseline to 12-months:** I:10.58 (6.29) *vs.* C: 11.08 (6.51)<br>**WT: change from baseline to 24-months:** I: 21.16 (10.04); C: 22.38 (11.92)<br><br>**N:**I: 186; C: 135<br>Also reports above for completers only.<br>Using the ITT model students in the intervention group significantly decreased their *z*BMI compared to students in the control group (−0.1; 95% CI: −0.1 to −0.02).<br><br>**Normal WT subgroup:**<br>Overall, 10.8% of students with a normal-WT status became overWT (10.4%) or obese (0.4%) at 24 months. No differences were found between the intervention (*n* = 239, completers) and control conditions (*n* = 137, completers) in terms of the likelihood of normal-WT students becoming overWT or obese compared to normal-WT control (OR: 1.66, NS).	There was significant improvement in weight but not zBMI in the South Asian overweight subgroup at 2 years follow-up. Study has internal validity but study sample may not be representative of study population as low response rate.

Table S3. *Cont.*

Study ID	Primary Outcomes	Summary
	There were no ethnic differences in terms of the likelihood of becoming overWT or obese for Asian students compared to Whites (OR: 1.40).<br><br>***Additional data provided by authors:***<br>**OverWT/obese SOUTH ASIAN subgroup:**<br>**BMI (kg/m^2): Change from baseline to 24-months:** I: 0.39 (0.74) *vs.* C: 0.78 (0.54)<br>**zBMI: Change from baseline to 24-months:** I: 0.01 (0.15) *vs.* C: 0.08 (0.17)<br>**WT: change from baseline to 24-months:** I: 4.20 (3.16); C: 6.79 (3.00)<br>**N:**I: 41; C: 15	
Kameswararao 2009 [15]	**Prevalence of obesity (BMI ≥ 95th percentile) baseline:** *n* = 59 children (9.67% of 610)<br>**Prevalence of obesity: (BMI ≥ 95th percentile) 6-months:** *n* = 57 of 59 children (9.34% of 610)	2 of 59 children were no longer obese after intervention. Study representativeness unclear.
Khaskheli 2013 [16]	**BMI (kg/m^2): Baseline:** 36.21 (1.35); **mean change at 6-months:** −9.6 (1.23);*n* = 85	BMI significantly reduced following intervention in obese infertile women. Small, selective study.
Kousar 2008 [17]	**BMI (kg/m^2): Baseline:**29.2 (0.46) *n* = 40;<br>**BMI (kg/m^2): week-12 (after control run-in):** 29.14 (0.46) *n* = 40;<br>**BMI (kg/m^2):24-weeks (after 12-week active intervention):**27.8 (0.45), *n* = 40	BMI significantly reduced following 12-weeks active intervention. Convenience sample specific to female Pakistani immigrant married women with children, living in Australia.
Madsen 2009 [18]	**% obese declined from 33% at baseline to 27% at follow up in total sample (*n* = 91)**<br>**Unadjusted change in BMI z-score at 8-months:** −0.04 all participants (*n* = 91) and −0.05 among obese participants (*n* = 29); −0.23 Asian participants (*n* = 17)<br>Also reports change in BMI z-score by Asian participants by normal/overWT/obese and by girls/boys. Also change in BMI (kg/m^2) in figure.	Response to the intervention differed significantly by race, with Asian children demonstrating the greatest decrease in BMI z-score. Study representativeness unclear. Lack of follow-up data for high proportion of sample.

Table S3. *Cont.*

Study ID	Primary Outcomes	Summary
Mathews 2007 [19]	**BMI (kg/m^2) baseline:** 28.60, *n* = 140<br>**BMI (kg/m^2) 6-month:** 28.30, *n* = 140<br>**Difference in BMI baseline to 6-month:** 0.30 (CI: 0.12 to 0.49)<br><br>**WC (cm) baseline:** 91.26 *n* = 126<br>**WC (cm) 6-month:** 90.75 *n* = 126<br>**Difference in WC baseline to 6-month:** 0.51 (CI: −0.3 to 1.3)<br><br>**WT (kg) baseline:** 70.77, *n* = 140<br>**WT (kg) 6-month:** 70.16, *n* = 140<br>**Difference in WT baseline to 6-month:** 0.61 (CI: 0.22 to 1.02)	WT and BMI significantly reduced at 6-month follow-up from baseline but not WC. Representative study sample but high attrition (completer analyses only) and unclear if outcomes attributable to the intervention.
Misra 2008 [20]	**BMI (kg/m^2) baseline:** 24.1 (3.9)<br>**BMI (kg/m^2) 3-month:** 24.1 (3.7)<br>**BMI (kg/m^2) 3-month difference:** 0.1 (1.1)<br><br>**WC (cm) baseline:** 87.9 (13.1)<br>**WC (cm) 3-month:** 86.3 (12.7)<br>**WC (cm) 3-month difference:** −1.6 (1.9)<br><br>**N:** 30	No significant change in BMI at 3-month follow-up. However, a significant decrease in WC was observed. Small selective sample.
Nidhi 2012 [21]	**BMI (kg/m^2) baseline:** I: 20.22 (1.65) *vs.* C: 21.28 (3.05)<br>**BMI (kg/m^2) 12 weeks:** I: 20.11 (1.74) *vs.* C: 21.59 (2.78)<br>**BMI change at 12-weeks:** I: 0.11 (0.51) *vs.* : 0.31 (1.63)<br><br>**WC (cm) baseline:** I: 0.66 (0.04) *vs.* C: 0.69 (0.07)<br>**WC (cm) 12 weeks:** I: 0.64 (0.03) *vs.* C: 0.69 (0.06)<br>**WC (cm) change at 12-weeks:** : 0.01 (0.03) *vs.* C: 0.006 (0.005)<br><br>**N:** I: 35; C: 36	Changes for BMI and WC were nonsignificant both within and between the groups. Study sample obtained from one residential school and control group weighed significantly more at baseline (unclear if this difference was accounted for in analyses).

Table S3. *Cont.*

Study ID	Primary Outcomes	Summary
Prabhakaran 2009 [22]	**WC (cm) baseline: I:** 84.3 (10.8); C: 86.5 (10.5)<br>**WC (cm) 3.7 to 4 years:** I: 79.9 (11.1); C: 85.2 (11.5)<br>**Adjusted mean difference in WC baseline to 3.7–4 years:** −3.5 (95% CI: −2.7 to −4.3)<br><br>**WT (kg) baseline:** 62.1 (12.8; C: 61.1 (10.2)<br>**WT (kg) 3.7 to 4 years:** 60.1 (13.8); C: 61.9 (11.0)<br>**Adjusted mean difference in WT baseline to 3.7–4 years:** −2.8 (95% CI: −2.1 to −3.5)<br><br>**N:** I: 1982; C: 349<br>Adjusted for baseline mean age, educational status, sex, baseline mean value of the dependent variable and BMI	Intervention group lost significantly more WT and WC compared to control. Cohort within repeat cross-sectional study. Intervention group of cohort more educated than cross-sectional intervention group (data not included in this review).
Ramachandran 2006 [23]	Significant increase in WT from baseline in the control group at 12, 24 and 30 months. A significant increase in WT was only seen in lifestyle intervention group at 24 months ($p = 0.035$).<br><br>***Additional data provided by authors:***<br>**BMI (kg/m^2): change at 30 months:** I: 0.1978 (1.3860) $n = 120$<br>**BMI (kg/m^2): change at 30 months: C:** 0.3589 (1.0601) $n = 133$<br>**WC: change at 30 months:** I: 0.8444 (6.119) $n = 120$<br>**WC: change at 30 months:** C: 0.9000 (5.681) $n = 133$<br>**N:** I: 120; C: 133	There was no significant difference between intervention and control groups for BMI or for WC at follow-up. High quality RCT.
Ramachandran 2013 [24]	**BMI (kg/m^2): Baseline;** I: 25.8 (3.3); C: 25.8 (3.0)<br>**BMI (kg/m^2): 20.2 months:** I: 25.0 (5.5); C: 25.0 (5.4);<br>**BMI (kg/m^2): Difference between I and C at 20.2 months (95% CI) adjusted for baseline differences and time:** I: −0.05 (−0.46 to 0.37)<br><br>**WC (cm): Baseline:** I: 92.6 (7.1); C: 92.7 (7.3);<br>**WC (cm): 20.2 months:** I: 92.6 (7.9); C: 92.6 (7.7);<br>**WC (cm): difference between I and C at 20.2 months (95% CI) adjusted for baseline differences and time:** I: 0.04 (−0.56 to 0.54)<br>**N:** I: 271; C: 266	No significant effect of intervention on BMI or WC. Good quality study but specific study sample: working men who owned a mobile phone.

Table S3. *Cont.*

**Study ID**	**Primary Outcomes**	**Summary**
Rush 2007 [25]	2 baseline measurements (1 month apart) were not significantly different and were therefore averaged and the average baseline values were compared with the post-intervention measurements separately by sex:<br>**WC (cm) baseline:** Female**:** 88.9 (9.0); Male: 95.6 (8.9)<br>**WC (cm) 5 months:** Female: 88.0 (8.8); Male: 93.6 (7.6)<br>**WC (cm) change at 5-months:** Female: −0.8 (3.5) Male: −2.0 (2.9)<br><br>**WT (kg) baseline:** Female**:** 63.3 (8.4); Male: 71.8 (10.2)<br>**WT (kg) 5 months:** Female**:** 62.1 (7.9); Male: 70.3 (9.8)<br>**WT (kg) change at 5-months:** Female: −1.2 (2.5) Male: −1.5 (1.8)<br><br>**N:** 41 (Female: 20 Male: 21)	WC and WT decreased significantly in men following the intervention while these changes were not statistically significant in women. Study sample recruited from community but response rate and attrition rate not reported.
Shailaja 2011 [26]	**URBAN BMI (kg/m^2) baseline:** Male: 28.09, *n* = 81; Female: 29.26, *n* = 19<br>**URBAN BMI (kg/m^2) 3-months:** Male: 26.96, *n* = 81; Female: 28.63, *n* = 19<br>**RURAL BMI (kg/m^2) baseline:** Male: 27.11, *n* = 46; Female: 28.18, *n* = 19<br>**RURAL BMI (kg/m^2) 3-months:** Male: 26.24, *n* = 46; Female: 26.71, *n* = 19<br><br>**URBAN WC (cm) baseline**: Male: 97.83, *n* = 81; Female: 92.56, *n* = 19<br>**URBAN WC (cm**) **3-months:** Male: 95.56, *n* = 81; Female: 91.50, *n* = 19<br>**RURAL WC (cm) baseline**: Male: 91.77, *n* = 46; Female: 94.29, *n* = 19<br>**RURAL WC (cm**) **3-months:** Male: 90.88, *n* = 46; Female: 93.22, *n* = 19<br><br>**URBAN WT (kg) baseline:** Male: 81.52, *n* = 81; Female: 72.21, *n* = 19<br>**URBAN WT (kg) 3-months:** Male: 78.38, *n* = 81; Female: 70.64, *n* = 19<br>**RURAL WT (kg) baseline:** Male: 72.53, *n* = 46; Female: 64.26, *n* = 17<br>**RURAL WT (kg) 3-months:** Male: 70.02, *n* = 46; Female: 61.58, *n* = 17	BMI and WC were significantly reduced at 3-month follow-up in both urban and rural males and females. Unclear if weight loss was significant. Intervention may have been confounded as participants advised to consult a dietician and fitness instructor to lose weight, and access to these services varied between participants. Unclear if study sample is representative of population; rural sample >50% attrition.

Table S3. *Cont.*

Study ID	Primary Outcomes	Summary
Sharma 2009 [27]	**BMI: Baseline:** Vatta (*n* = 55) 30.97(5.32); Pitta (*n* = 83) 31.98(5.21); Kapha (*n* = 62) 31.63(4.95)<br>**BMI: 3 month:** Vatta (*n* = 55) 28.01(4.98); Pitta (*n* = 83) 28.83(4.50); Kapha (*n* = 62) 28.63(4.33)<br>**WC (cm): baseline:** Vatta: 92.74 (14.80) *n* = 55; Pitta: 93.50 (13.97) *n* = 83; Kapha: 93.15 (10.09) *n* = 62;<br>**WC (cm): 3 months:** Vatta: 85.24 (13.93) *n* = 55; Pitta: 84.45 (13.17) *n* = 83; Kapha: 85.72 (9.81) *n* = 62;<br>**WC (cm) % change at 3-months**: Vatta: −8.1% *n* = 55; Pitta: −9.7% *n* = 83; Kapha: −8.0% *n* = 62;<br>**WT (kg): Baseline:**<br>Vatta (*n* = 55) 81.14(19.23); Pitta (*n* = 83) 83.78(18.52); Kapha (*n* = 62) 4.65(16.29)<br>**WT (kg): 3 months:**<br>Vatta (*n* = 55) 73.41(17.65); Pitta (*n* = 83) 75.54(16.96); Kapha (*n* = 62) 76.53(13.71)<br>**WT (kg) change at 3-months:**<br>Vatta: −7.73 (9.53%), *n* = 55; Pitta: −8.24 (9.84%) *n* = 83; Kapha: −8.12 (9.59%) *n* = 62	All groups significantly reduced BMI, WC and WT; the *pitta* group lost the most WT (9.84%). Study sample unlikely to be representative of general population and significant baseline differences between groups for WT. Low quality study.
Singhal 2010 [28]	**BMI (kg/m^2) change at 6-months:** I: −0.07 (0.71) *vs.* C: −0.06 (1.11)<br>**BMI (kg/m^2) between group difference at 6-months: 95% CI:** −0.18 to 0.34<br>**WC (cm) change at 6-months:** I: −0.65 (3.99) *vs.* C: 0.65 (4.15)<br>**WC (cm) between group difference at 6-months: 95% CI:** −2.43 to −0.17<br>**WT (kg) change at 6-months:** I: 1.52 (10.52) *vs.* C: 0.69 (2.5)<br>**WT (kg) between group difference at 6-months: 95% CI:** −2.94 to 1.28<br>**N:** 201 (I:99 *vs.* C:102)	No significant between group differences for BMI or WT, however significant reduction in WC in intervention schools compared to control schools. Two matched schools representing middle SES adolescents.
Telle-Hjellset 2013 [29]	**BMI (kg/m^2): Baseline (95% CI):** I: 29.39 (28.2 to 30.6) *n* = 91; C: 29.73 (28.6 to 30.9) *n* = 86;<br>**BMI (kg/m^2): 7-month (95% CI):** I: 29.11 (28.0 to 30.3) *n* = 91; C: 29.65 (28.5 to 30.8) *n* = 86;<br>**BMI (kg/m^2): 7-month change (95% CI):** I: −0.28 (−0.50 to −0.06) *n* = 91; C: −0.08 (−0.28 to 0.11) *n* = 86 −0.28 (−0.50, −0.06)<br>When completers in control group compared to participants in intervention group who attended at least 4 group sessions, there was a significant difference between groups at follow-up for BMI in favour of the intervention group (−0·48, 95% CI 20·78 to 20·18) kg/m2). Changes in BMI were not significantly related to SES.<br>**WC (cm): Baseline (95% CI):** I: 95.3 (92.4 to 98.1) *n* = 89; C: 96.2 (93.9 to 98.5) *n* = 86;<br>**WC (cm): 7-months (95% CI):** I: 95.1 (92.4 to 97.9) *n* = 89; C: 96.8 (94.5 to 99.1) *n* = 86;<br>**WC (cm): 7-months change (95% CI):** I: −0.14 (−0.84 to 0.56) *n* = 91; C: 0.58 (−0.24 to 1.40) *n* = 86<br>**WT (kg) change at 6-months (mean, min, max):** I: −0.77 (−10.15, 4.95) *n* = 82; C: −0.27 (−9.30, 4.35) *n* = 77	BMI significantly reduced from baseline to follow-up in intervention group but not significantly different between groups at follow-up. No significant change in WC. Average weight change was not significantly different between intervention and control groups. High quality RCT.<br>Assessed outcomes by SES and found improvement in BMI in intervention group was not affected by SES; low-SES were reached by the intervention.

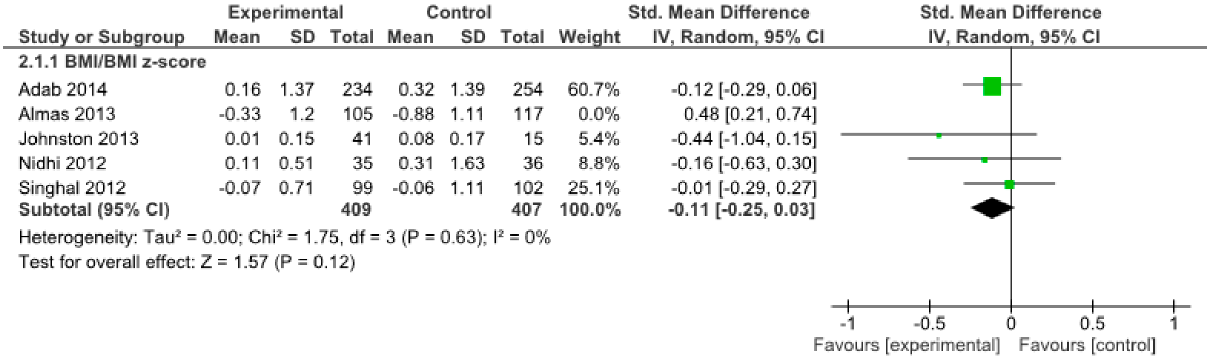

Figure S2. Meta-analysis of mean change in BMI/zBMI from baseline to post intervention for South Asian Children, sensitivity analysis excluding outlying study.

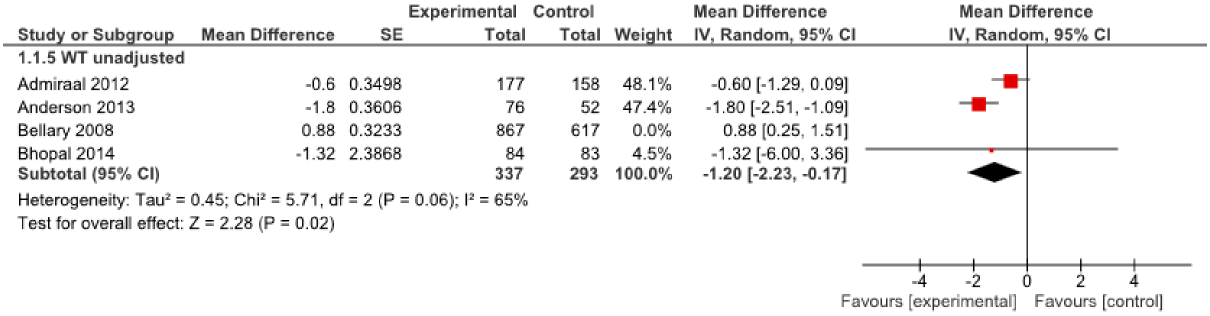

Figure S3. Meta-analysis of mean change in weight from baseline to post intervention for South Asian adults, sensitivity analysis excluding outlying study.

Table S4. Meta regression analysis of diet and physical activity interventions among South Asian children and adults.

Variable	Pooled Mean Difference	I^2 (95% CI)	Q-Value (DF)	*p*-Value
Pooled Effect	−0.42 (−0.62; −0.23)	86.3% (82%; 89.6%)	3248.66 (34)	<0.000
Meta-regression results (R^2 = 74.77% & I^2 = 51.64%)				
Variable	Estimate	SE	Z-value	Lower
Intercept	−0.15	0.26	−0.59	0.553
Outcome(ref = BMI)				
WC	−0.77	0.20	−3.80	0.000
WT	−0.35	0.23	−1.52	0.129
Group(ref = Adult)				
Child	0.03	0.34	0.10	0.924
Outcome* Group	0.72	0.31	2.32	0.020
Author(ref = Adab 2014 [1], Telle-Hjellset 2013 [29])				
Admiraal 2013 [2]	−0.06	0.35	−0.18	0.861
Anderson 2013 [4]	−1.05	0.31	−3.41	0.001
Bellary 2008 [8]	0.70	0.30	2.36	0.018
Bhopal 2014 [9]	−0.62	0.37	−1.71	0.087

Table S4. *Cont.*

Variable	Pooled Mean Difference	I^2 (95% CI)	Q-Value (DF)	*p*-Value
Johnston 2013 [14]	−0.32	0.45	−0.72	0.469
Nidhi 2012 [21]	0.16	0.30	0.52	0.601
Ramachandran 2006 [23], 2013 [24]	0.30	0.30	1.01	0.314
Singhal 2010 [28]	−0.02	0.27	−0.06	0.949

Notes: all data were combined with additional three variables created to differentiate between the different studies. The variable "Group" was used to differentiate between children and adult studies whilst variable "Outcome" was used to differentiate between the different outcome types, body mass index (BMI), waist circumference (WC) and weight (WT). We also created a variable for authors' identification since there were 11 authors contributing thirty-five data points. The pooled effect from a random effect model was significantly different from zero, but with substantial evidence of heterogeneity between the studies. Heterogeneity between studies explained about 86% of the total variability in the model. In order to explain the observed heterogeneity in the data, we fitted a meta-regression model with authors, outcome, group and the interaction between group and outcome types as moderators. Test of moderators was statistically significant (QM (df = 12) = 91.57, *p*-value < 0.0001). The proportion of variability explained by the moderators was 74.8% (R^2). The results also showed significant interaction effects between the outcome types and the group. There was a significant association between authors' identification and the intervention effects, which maybe an indication of differential reporting or varying quality in the reported data.

References

1. Adab, P.; Pallan, J.; Cade, J.; Ekelund, U.; Barrett, T.; Daley, A.; Deeks, J.; Duda, J.; Gill, P.; Parry, J.; *et al*. Preventing childhood obesity, phase II feasibility study focusing on South Asians: BEACHeS. *BMJ Open* **2014**, *4*, doi:10.1136/bmjopen-2013-004579
2. Admiraal, W.M.; Vlaar, E.M.; Nierkens, V.; Holleman, F.; Middelkoop, B.J.C.; Stronks, K.; van Valkengoed, I.G.M. Intensive lifestyle intervention in general practice to prevent type 2 diabetes among 18 to 60-year-old South Asians: 1-Year effects on the weight status and metabolic profile of participants in a randomized controlled trial. *PLoS One* **2013**, *8*, doi:10.1371/journal.pone.0068605.
3. Almas, A.; Islam, M.; Jafar, T.H. School-based physical activity programme in preadolescent girls (9–11 years): A feasibility trial in Karachi, Pakistan. *Arch. Dis. Child* **2013**, *98*, 515–519.
4. Andersen, E.; Hostmark, A.T.; Holme, I.; Anderssen, S.A. Intervention effects on physical activity and insulin levels in men of Pakistani origin living in Oslo: A randomised controlled trial. *J. Immigr. Minor. Health* **2013**, *15*, 101–110.
5. Backes, A.C.; Abbasi, F.; Lamendola, C.; McLaughlin, T.L.; Reaven, G.; Palaniappan, L.P. Clinical experience with a relatively low carbohydrate, calorie-restricted diet improves insulin sensitivity and associated metabolic abnormalities in overweight, insulin resistant South Asian Indian women. *Asia Pac. J. Clin. Nutr.* **2008**, *17*, 669–671.
6. Balagopal, P.; Kamalamma, N.; Patel, T.G.; Misra, R. A community-based diabetes prevention and management education program in a rural village in India. *Diabetes Care* **2008**, *31*, 1097–1104.

7. Balagopal, P.; Kamalamma, N.; Patel, T.G.; Misra, R. A community-based participatory diabetes prevention and management intervention in rural India using community health workers. *Diabetes Educ*. **2012**, *38*, 822–834.
8. Bellary, S.; Raymond, N.T.; Gumbar, A.; Mughal, S.; Szczepura, A.; Kumar, S.; Barnett, A.H.; UKADS Study Group. Enhanced diabetes care to patients of south Asian ethnic origin (The United Kingdom Asian Diabetes Study): A cluster randomised controlled trial. *Lancet* **2008**, *371*, 1769–1776.
9. Bhopal, R.S.; Douglas, A.; Wallia, S.; Forbes, J.F.; Lean, M.E.J.; Gill, J.M.R.; McKnight, J.A.; Sattar, N.; Sheikh, A.; Wild, S.H.; *et al*. Effect of a lifestyle intervention on weight change in South Asian individuals in the UK at high risk of type 2 diabetes: A family-cluster randomised controlled trial. *Lancet Diabetes Endocrinol.* **2014**, *2*, 218–227.
10. Chander, J.S.J.U.; Prakasam, A.; Kannan, S.; Kumar, S.; Tyagi, M.G. A study of pharmaceutical care impact on cardiovascular risk in diabetic and hypertensive patients. *Int. J. Pharm. Sci. Res.* **2013**, *4*, 3135–3141.
11. Dixon, A.N.; Valsamakis, G.; Hanif, M.W.; Field, A.; Boutsiadis, A.; Harte, A.; McTernan, P.G.; Barnett, A.H.; Kumar, S. Effect of the orlistat on serum endotoxin lipopolysaccharide and adipocytokines in South Asian individuals with impaired glucose tolerance. *Int. J. Clin. Pract.* **2008**, *62*, 1124–1129.
12. Ghosh, A. Effects of daily exercise on blood pressure, plasma glucose, and obesity measures in 55–64-year-old obese Asian Indian men: The Calcutta longitudinal study. *Amer. J. Hum. Biol.* **2006**, *18*, 718–721.
13. Gulati, S.G.; Misra, A.; Pandey, R.M.; Bhatt, S.P.; Saluja, S. Effects of pistachio nuts on body composition, metabolic, inflammatory and oxidative stress parameters in Asian Indians with metabolic syndrome: A 24-week, randomized control trial. *Nutrition* **2014**, *30*, 192–197.
14. Johnston, C.A.; Moreno, J.P.; El-Mubasher, A.; Gallagher, M.; Tyler, C.; Woehler, D. Impact of a school-based pediatric obesity prevention program facilitated by health professionals. *J. Sch. Health* **2013**, *83*, 171–181.
15. Kameswararao, A.A.; Bachu, A. Survey of childhood diabetes and impact of school level educational interventions in rural schools in Karimnagar district. *Int. J. Diabetes Dev. Ctries* **2009**, *29*, 69–73.
16. Khaskheli, M.N.; Baloch, S.; Baloch, A.S. Infertility and weight reduction: Influence and outcome. *J. College Phys. Surg. Pak.* **2013**, *23*, 798–801.
17. Kousar, R.; Burns, C.; Lewandowski, P. A culturally appropriate diet and lifestyle intervention can successfully treat the components of metabolic syndrome in female Pakistani immigrants residing in Melbourne, Australia. *Metabolism* **2008**, *57*, 1502–1508.
18. Madsen, M.A.; Thompson, H.R.; Wlasiuk, L.; Queliza, E.; Schmidt, C.; Newman, T.B. After-school program to reduce obesity in minority children: A pilot study. *J. Child Health Care* **2009**, *13*, 333–346.
19. Mathews, G.; Alexander, J.; Rahemtulla, T.; Bhopal, R.; Mathews, G.; Alexander, J.; Rahemtulla, T.; Bhopal, R. Impact of a cardiovascular risk control project for South Asians (Khush Dil) on motivation, behaviour, obesity, blood pressure and lipids. *J. Public Health* **2007**, *29*, 388–397.

20. Misra, A.; Alappan, N.K.; Vikram, N.K.; Goel, K.; Gupta, N.; Mittal, K.; Bhatt, S.; Luthra, K. Effect of supervised progressive resistance-exercise training protocol on insulin sensitivity, glycemia, lipids, and body composition in asian indians with type 2 diabetes. *Diabetes Care* **2008**, *31*, 1282–1287.
21. Nidhi, R.; Padmalatha, V.; Nagarathna, R.; Ram, A. Effect of a yoga program on glucose metabolism and blood lipid levels in adolescent girls with polycystic ovary syndrome. *Int. J. Gynaecol. Obstet.* **2012**, *118*, 37–41.
22. Prabhakaran, D.; Jeemon, P.; Goenka, S.; Lakshmy, R.; Thankappan, K.; Ahmed, F.; Joshi, P.; Mohan, B.; Meera, R.; Das, M.; *et al*. Impact of a worksite intervention progam on cardiovascular risk factors. *J. Amer. Coll. Cardiol.* **2009**, *53*, 1718–1728.
23. Ramachandran, A.; Snehalatha, C.; Mary, S.; Mukesh, B.; Bhaskar, A.; Vijay, V.; Indian Diabetes Prevention Programme (IDPP). The Indian diabetes prevention programme shows that lifestyle modification and metformin prevent type 2 diabetes in Asian Indian subjects with impaired glucose tolerance (IDPP-1). *Diabetologia* **2006**, *49*, 289–297.
24. Ramachandran, A.; Snehalatha, C.; Ram, J.; Selvam, S.; Simon, M.; Nanditha, A.; Shetty, A.S.; Godsland, I.F.; Chaturvedi, N.; Majeed, A.; *et al*. Effectiveness of mobile phone messaging in prevention of type 2 diabetes by lifestyle modifi cation in men in India: A prospective, parallel-group, randomised controlled trial. *Lancet Diabetes Endocrinol.* **2013**, *1*, 191–198.
25. Rush, E.C.; Chandu, V.; Plank, L.D. Reduction of abdominal fat and chronic disease factors by lifestyle change in migrant Asian Indians older than 50 years. *Asia Pac. J. Clin. Nutr.* **2007**, *16*, 671–676.
26. Shailaja, K.; Moideen, M.M.; Kumar, M.A.; Ramasamy, C. Effectiveness of patient counseling on weight reduction in rural and urban overweight and obese patients. *Int. J. Pharma Bio Sci.* **2011**, *2*, 173–185.
27. Sharma, S.; Puri, S.; Agarwal, T.; Sharma, V. Diets based on Ayurvedic constitution--potential for weight management. *Altern. Ther. Health Med.* **2009**, *15*, 44–47.
28. Singhal, N.; Misra, A.; Shah, P.; Gulati, S. Effects of controlled school-based multi-component model of nutrition and lifestyle interventions on behavior modification, anthropometry and metabolic risk profile of urban Asian Indian adolescents in North India. *Eur. J. Clin. Nutr.* **2010**, *64*, 364–373.
29. Telle-Hjellset, V.; Raberg Kjollesdal, M.K.; Bjorge, B.; Holmboe-Ottesen, G.; Wandel, M.; Birkeland, K.I.; Eriksen, H.R.; Hostmark, A.T. The InnvaDiab-DE-PLAN study: A randomised controlled trial with a culturally adapted education programme improved the risk profile for type 2 diabetes in Pakistani immigrant women. *Brit. J. Nutr.* **2013**, *109*, 529–538.

© 2015 by the authors; licensee MDPI, Basel, Switzerland. This article is an open access article distributed under the terms and conditions of the Creative Commons Attribution license (http://creativecommons.org/licenses/by/4.0/).
